# Flash-kinetics as a complementary analytical tool in PAM fluorimetry

**DOI:** 10.1007/s11120-024-01101-w

**Published:** 2024-05-22

**Authors:** Christof Klughammer, Friedemann Schlosser, Ulrich Schreiber

**Affiliations:** 1 79618 Rheinfelden, Germany; 2 97080 Würzburg, Germany; 3 Julius-von-Sachs Institut für Biowissenschaften, Universität Würzburg, Julius-von-Sachs Platz 2, 97082 Würzburg, Germany

**Keywords:** MULTI-COLOR-PAM, Polyphasic fluorescence rise O-I_1_-I_2_-P, Single and multiple turnover flashes, Carotenoid triplet quenching, Donor-side dependent quenching, Period-4 oscillations

## Abstract

A new measuring system based on the already existing Multi-Color-PAM Fluorimeter (Schreiber et al. in Photosynth Res 113:127–144, 2012) was developed that in addition to standard PAM measurements enables pump-and-probe flash measurements and allows simultaneous measurements of the changes in chlorophyll fluorescence yield (F) *during* application of saturating flashes (ST). A high-power Chip-on-Board LED array provides ST flashes with close to rectangular profiles at wide ranges of widths (0.5 µs to 5 ms), intensities (1.3 mmol to 1.3 mol 440 nm quanta m^−2^ s^−1^) and highly flexible repetition times. Using a dedicated rising-edge profile correction, sub-µs time resolution is obtained for assessment of initial fluorescence and rise kinetics. At maximal to moderate flash intensities the flash-kinetics (changes of F *during* course of ST, STK) are strongly affected by ‘High Intensity Quenching’ (HIQ), consisting of Car-triplet quenching, TQ, and donor-side-dependent quenching, DQ. The contribution of TQ is estimated by application of a second ST after 20 µs dark-time. Upon application of flash trains (ST sequences with defined repetition times) typical period-4 oscillations in dark fluorescence yield (F_0_) and ST-induced fluorescence yield, F_m_^ST^, are obtained which can be measured in vivo both with suspensions and from the surface of leaves. Examples of application with dilute suspensions of *Chlorella* and an intact dandelion leaf are presented. It is shown that weak far-red light (730–740 nm) advances the S-state distribution of the water-splitting system by one step, resulting in substantial lowering of F_m_^ST^ and also of the I_1_-level in the polyphasic rise of fluorescence yield induced by a multiple-turnover flash (MT). Based on comparative measurements of STK and the polyphasic rise kinetics with the same *Chlorella* sample, it is concluded that the generally observed lower values of maximal fluorescence yields using ST-protocols compared to MT-protocols are due to a higher extent of HIQ (mainly DQ) and the contribution of variable PSI fluorescence to F_m_^ST^.

**Supplementary Information:**

The online version contains supplementary material available at 10.1007/s11120-024-01101-w.

## Introduction

Single turnover flashes (ST) have been playing an outstanding role in photosynthesis research and in the study of chlorophyll (Chl) fluorescence in particular. Upon application of an ST, a charge separation in photosystem II (PSII) is induced by which the primary acceptor Q_A_ is reduced and the primary donor P680 is oxidized (formation of the state Q_A_^−^P680^+^). Notably, formation of this state involves disappearance of the photochemical fluorescence quencher Q_A_, and appearance of the non-photochemical quencher P680^+^, both of which cause quenching of variable fluorescence (Okayama and Butler 1971, Mauzerall 1972, den Haan et al. 1974, Sonneveld et al. 1979, Shinkarev and Govindjee 1993, Reifarth et al. 1997, Christen et al. 1999, Steffen et al. 2001, Lazar 2003, Zhu et al. 2005, Belyaeva et al. 2008). Following ST-induced primary charge separation in PSII, there is a cascade of secondary dark reactions in the ns to ms time range, by which the positive and negative charges are stabilized at the PSII donor and acceptor sides, respectively. The flash induced changes in fluorescence yield are dominated by the fact that reduction of Q_A_ is quasi-instantaneous, whereas stabilization of the positive charge occurs in the ns to ms time range, depending on the pregiven state of the oxygen evolving complex, OEC. Hence, ST-induced changes of fluorescence yield in the ns to ms time range *cannot* simply be interpreted by changes of Q_A_^−^, as originally suggested by Duysens and Sweers (1963) for fluorescence changes induced by moderate continuous light in the ms to s time range. The same is also true for the rapid changes of fluorescence yield induced by saturating multiple turnover pulses of light (MT) that are used in PAM fluorimetry for measuring the polyphasic rise kinetics (O-I_1_-I_2_-P or O-J-I-P) of fluorescence yield (Schreiber 1986; Schreiber 2004, for an extensive review see Stirbet and Govindjee 2012). In this case, when more than one charge separation is induced, in addition also the secondary electron transfer reactions at the PSII *acceptor side* play a role, i.e. the rates with which Q_A_^−^ is reoxidized by Q_B_ and Q_B_^−^, which initially (i.e. as long as oxidized PQ is available to bind to the Q_B_ binding site) are faster than the slowest step of the OEC cycle (for a recent review see Shevela et al. 2023). In general, whenever the rate of PSII charge separation exceeds the rate of charge stabilization at the donor-side (i.e. of S-state advancement), donor-side dependent quenching of fluorescence yield (DQ) should be expected, i.e. the measured fluorescence yield will be lower than maximal fluorescence yield, in spite of Q_A_ being fully reduced and photochemical energy conversion in PSII being blocked.

Intensive studies of ST-induced Chl fluorescence changes were triggered more than 50 years ago by the discovery of period-4 oscillations in fluorescence yield as a function of the number of ST in a train of ST separated by short dark times. Joliot et al. (1971) and Delosme (1971a, b) discovered period-4 oscillations in the initial fluorescence yield, F_0_, and in maximal fluorescence yield, F_m_^ST^, respectively. The oscillation patterns were found to closely relate to that of the yield of flash-induced oxygen evolution in a series of ST, which could be satisfactorily interpreted by the 4-step charge accumulation model of water-splitting proposed by Kok et al. (1970). The fluorescence data suggested that F_0_ follows the sum of the S_0_ + S_1_ population and F_m_^ST^ that of the S_2_ + S_3_ population briefly before an ST. Van Gorkom and Donze (1973) presented a molecular model for the control of fluorescence yield by the local electric field that is caused by positive charges on various components at the PSII donor side and on the efficiency of charge separation. At the present stand of information, a major role of the oxidized primary donor P680^+^ in equilibrium with its donor tyrosine Z (Yz) and the S-states of the OEC may be assumed (Schilstra et al. 1998; Christen et al. 1999). In this context, the S_3_ state is most important, as after charge separation in the S_3_ state (driven by an ST or an MT) stabilization via S_4_ and S_0_ formation is exceptionally slow (half-time of 1.3 ms, as compared to 30 µs in the case of S_0_) (Dau and Haumann 2007).

Since the recognition that ST-induced fluorescence changes provide detailed information on the mechanism of water-splitting at high time resolution, considerable efforts have been made to measure such changes as close as possible to ST-off or even *during* the ST. Delosme (1971a,b) applied the same saturating 2.5 µs xenon-flash for inducing PSII turnover and for integrative measurement of the fluorescence yield in the charge-separated state (see section on ‘Sequences of ST-kinetics and period-4 oscillations: Delosme (1971) revisited’ below). Zankel (1973) was the first to measure the *kinetics* of fluorescence changes *during* a saturating 2 µs xenon-flash, consisting of a rapid rise to a peak that was followed by a decline to a stationary level. He identified the latter to reflect the light driven formation of carotenoid triplets that quench Chl fluorescence. This ‘triplet quenching’ (TQ), in addition to donor-side dependent quenching (DQ), essentially complicates the analysis of ST-induced fluorescence changes, both in terms of assessment of the rate of ST-driven Q_A_ reduction (increase of fluorescence yield) and the post-ST rate of Q_A_ reoxidation (decrease of fluorescence yield). Consequently, in most studies of ST-induced fluorescence changes, these have been measured at least 20 µs after ST-off, when TQ is mostly relaxed.

When the ST-induced fluorescence changes are measured *during* an ST, it *cannot be avoided* that both TQ and DQ are effective. In this case, a relatively low ST-intensity can be chosen in order to minimize TQ, which increases with the excitation density (Zankel 1973; Breton et al. 1979; Sonneveld et al. 1979; Schreiber et al. 2021). Alternatively, instead of a single strong ST a rapid sequence of many less strong ‘flashlets’ with short dark-times in between can be applied, assuming that at least part of TQ will relax during the dark periods (Kolber et al. 1998; Gorbunov et al. 1999; Ananyev and Dismukes 2005). In any case, however, even when TQ is minimized, lowering of the maximal fluorescence yield measured during an ST *by DQ* cannot be avoided. In case of a relatively low effective ST intensity, a correspondingly larger ST width is required for complete Q_A_ reduction. Longer ST, however, may lead to double turnovers, that inevitably will stimulate DQ, as the lifetime of the non-photochemical quencher P680^+^ increases with transition to higher S-states. In this context, it has to be considered that recombination between Q_A_^−^ and P680^+^ will occur whenever the lifetime of P680^+^ is extended into the 100 µs range, which is the case upon inactivation of the OEC (Havemann and Mathis 1976, Renger and Wolff 1976). While formally the resulting fluorescence quenching is photochemical, it is “dissipative” and, therefore, falls into the same category as non-photochemical donor-side dependent quenching, DQ. These aspects bear on the observed differences between the maximal fluorescence yields measured by ST- as well as MT-protocols and have to be taken into account in the evaluation of recent findings on the requirement of numerous saturating ST for reaching maximal fluorescence yield in the presence of DCMU (Magyar et al. 2018; Sipka et al. 2019; Garab et al. 2023).

In PAM fluorimetry (Schreiber et al. 1986) maximal fluorescence yield traditionally is determined by an MT-protocol, i.e. using an about 300 ms long pulse of saturating light that leads to full reduction not only of Q_A_, but of the secondary acceptor pools of PSII and PSI as well. With charge separation in PSII being completely blocked, the formation of higher S-states is prevented and the donor-side can relax into a state similar to the dark-state. However, F_m_ still can be quenched by TQ which in the range of commonly applied MT-intensities is proportional to MT-intensity (Schreiber et al. 2021). Therefore, unnecessarily high MT-intensity should be avoided.

Here we report on a new measuring system that allows comparative and even simultaneous measurements of the yield of Chl fluorescence using ST- and MT-protocols on the same sample and in the same optical geometry. We have developed the means to measure the fluorescence yield in dilute suspensions as well as in intact leaves with sub-µs time resolution *during* an ST (ST-Kinetics, STK) as well as at variable dark-times after ST-off (pump-and-probe approach). Furthermore, the new device allows profound analysis of flash-train responses (STK sequences, STKS, which display period-4 oscillations in physiologically healthy samples), thus providing a powerful new tool for the study of reactions at the PSII donor-side. A very satisfactory signal/noise ratio is obtained without signal averaging, which is of great practical value for in vivo applications, in which the reproduction of a particular state for signal averaging often is difficult and time consuming. We will show that the long-standing experimental fact, of F_m_^ST^ < F_m_^MT^ in vivo can be at least partially explained by.

1. lowering of F_m_^ST^ by DQ and
2. a F_v_(I) component that is induced by an MT at the end of the polyphasic rise (Schreiber et al. 2021; Schreiber 2023).

Some examples of applications will be presented that demonstrate the performance of the new measuring system in the asssessment of period-4 oscillations of DQ in a dilute suspension of *Chlorella* and an intact dandelion leaf, with emphasis on a surprisingly strong effect of very weak far-red light on the initial S-state distribution.

## Materials and methods

### Photosynthetic material and sample preparation

Most experiments were carried out with dilute suspensions of green unicellular algae *Chlorella vulgaris* (SAG 211-11b). *Chlorell*a was cultured in natural day light (north window) at 20–40 µmol m^−2^ s^−1^ and ambient temperature (20–25 °C) in BG11 medium under ambient air. The batch culture was shaken manually at least 4 times per day and frequently diluted so that the chlorophyll (Chl) content did not exceed 5 mg  l^−1^. All experiments were carried out at room temperature (22–25 °C) with the stock suspension diluted by the BG11 medium to final Chl concentrations of 200–500 µg l^−1^, as determined with a calibrated WATER-PAM chlorophyll fluorimeter (Heinz Walz GmbH, Effeltrich, Germany). 1.3 ml of dilute suspension was continuously stirred within the 10 mm × 10 mm cuvette in the standard Optical Unit ED-101US (Walz) with the help of a small magnetic “flea”. During Fast Kinetics recordings stirring was automatically interrupted for a couple of seconds, controlled by the user software (PamWin-4 program).

Particular attention was paid to avoid inadvertent and uncontrolled preillumination of samples by ambient light. For this purpose, in addition to the standard hood on top of the cuvette, the whole Optical Unit was covered with a black cloth. As will be outlined below (see sections on ‘Comparison of ST-kinetics (STK) and MT-induced polyphasic rise kinetics measured with the same dilute suspension of *Chlorella’*, *‘*S-state advancement induced by weak FR in *Chlorella’* and ‘S-state advancement induced by weak FR in a dandelion leaf’), the observed flash-induced responses are strongly influenced by surprisingly low intensities of background light. For the measurement of Fig. 14 a light green dandelion leaf (Taraxacum officinale) was used that was collected from a shaded garden habitat in Würzburg.

### Terminology of combined PAM and ST-kinetics measurements

The new measuring system constitutes an extension of the already existing Multi-Color-PAM fluorimeter that was introduced more than 10 years ago (Schreiber et al. 2012). In addition to assessment of Chl fluorescence yield using weak, multi-wavelength pulse modulated measuring light (ML), it also allows time resolved measurements of fluorescence directly excited by very strong pulses of actinic light, including saturating µs-flashes. Hence, extremely different levels of fluorescence intensity are involved. Nevertheless, for the sake of continuity, as far as possible the terminology that has been used with PAM fluorimetry shall be maintained.

In PAM fluorimetry the measured fluorescence *intensity* can be treated as an indicator of fluorescence *yield*, as the fluorescence is excited by pulse-modulated measuring light of *constant intensity* that by itself does *not* change the state of the photosynthetic apparatus. Changes in the latter are induced by non-modulated actinic light (AL), multiple-turnover pulses of light (MT) or single-turnover flashes (ST). The dark level fluorescence (F_0_), with all PSII reaction centers being open, is assessed by the ML at low ML-pulse frequency. Maximal fluorescence yield, F_m_, is measured during application of a saturating MT (or Saturation Pulse, SP) which leads to full closure of PSII reaction centers (RCII). Notably, although an MT may excite 10^6^ times more fluorescence than the time integrated ML, the measured pulse-modulated fluorescence intensity does not vary by much more than a factor of 5, corresponding to a ratio of variable fluorescence (F_v_) to F_m_ of 0.8. SP-quenching analysis (for a review, see Schreiber 2004) allows to differentiate between various types of energy losses. While this analysis is most informative under conditions of continuous, steady-state illumination, measurements of the polyphasic fluorescence rise kinetics upon onset of strong actinic illumination are most informative after dark-adaptation, featuring the characteristic fluorescence levels O, I_1_, I_2_ and P (terminology originally introduced for PAM fluorimetry, Schreiber 1986).

Measurements of flash-kinetics (or ST-Kinetics, STK) with the new instrument differ from PAM measurements in that the assessed fluorescence is *directly* excited by the flash and no separate ML is involved. While in PAM fluorimetry the width of individual ML-pulses is constant at about 1 µs and the time span of light induced changes is determined by the AL- or MT-width, the STK-time span is largely variable in the µs to ms time range, being determined by the ST-width. In spite of these basic differences, PAM and STK data are quantitatively comparable in terms of *relative fluorescence yield* (see section below on ‘Quantitative comparison of the relative fluorescence yields measured via PAM fluorimetry and ST-kinetics (STK)’).

In a given physiological state, fluorescence *intensity* is proportional to the intensity of excitation. The relative fluorescence yield is determined by the ratio of measured fluorescence intensity divided by the intensity of excitation. This means that after normalization of the fluorescence intensities measured by PAM and STK in a given state of the sample, any changes in the relative fluorescence yields measured by the two techniques are equivalent. In practice, normalization preferentially is carried out in the dark-adapted state. After such normalization, the initial fluorescence intensity (F_i_) of the STK and the fluorescence intensity in weak ML (F_0_) are equal and the relative fluorescence yields assessed by the two techniques remain equivalent, as long as the PAM ML intensity and the ST-intensity are not changed.

ST flashes employed by standard PAM fluorimeters, like the Multi-Color-PAM, have been limited to a fixed maximal intensity, with the actinic effect being controlled by the ST-width (ranging between 2.5 to 50 µs). In this case, the definition of an ST as a single turnover flash is straight forward. Whether it is saturating or not (in terms of Q_A_ reduction) depends on its width and the effective PSII absorption cross-section of the sample. With the new measuring system, substantially longer flashes with a large range of intensities are available, which may allow more than one turnover of RCII. Nonetheless the new flash-source will be referred to as an “ST-lamp” and the term “ST-kinetics” (STK) will be used for all measurements in which changes of fluorescence yield are induced by the”ST-flashes” generated by this lamp.

In PAM measurements with dilute suspensions of chloroplasts, algae and cyanobacteria, relative fluorescence yield is increased to the so-called I_1_-level upon application of a saturating ST. This corresponds to the first intermediate level of fluorescence yield between F_0_ and F_m_ observed in the polyphasic rise kinetics induced upon application of strong actinic light after dark adaptation (Schreiber 1986). As will be shown below, the fluorescence yield measured at the end of an STK (i.e. *during* application of a saturating ST) tends to be lowered with respect to the I_1_-level, due to non-photochemical quenching (so-called high-energy-quenching, HIQ) which increases with ST-intensity.

### Key components for combined PAM and ST-kinetics measurements

The experimental setup for combined PAM and ST-kinetics measurements essentially consists of an updated version of the Multi-Color-PAM Chlorophyll Fluorometer developed by Christof Klughammer, Jörg Kolbowski and Ulrich Schreiber (since 2011 commercially available via Heinz Walz GmbH, Germany) and a novel *ST-lamp* combined with two types of ST-signal detectors, developed by the authors. Technical features of the Multi-Color-PAM were previously described in detail (Schreiber et al. 2012; Schreiber and Klughammer 2021; Schreiber 2023). This instrument is particularly well suited for measuring rapid fluorescence changes in suspensions of algae and cyanobacteria, with variation of the wavelengths of excitation and emission, as well as of the colors of the actinic light that drives the changes of fluorescence yield. It combines high sensitivity with high time resolution. The standard version provides pulse-modulated measuring light (ML) at 400, 440, 480, 540, 590 and 625 nm. For improvement of performance in conjunction with the new components for measuring ST-kinetics, the drivers of the various LED light sources were integrated into the Emitter-unit (MCP-E II). Furthermore, the control unit (MCP-C II) now features a microprocessor with 60 × higher clock rate and three 14-bit AD-converters, facilitating simultaneous recording of two independent PAM signals (i.e. using two separate PAM detectors) at a sampling rate of 400 kHz. In addition, the control unit includes a separate high speed (40 MHz) 14-bit AD-converter for digitizing the rapid ST-signals or any other external analog signals. Data transfer between the instrument and the computer is speeded up using USB 2.0.

#### ST-Emitter-Detector unit (ST-lamp)

At the core of the ST-Emitter-Detector unit (ST-lamp) is a custom-made chip on board (COB) light-emitting diode (LED) array consisting of 22 blue (440 nm) Power-LED chips (type C4L-D47X2, Chips-4-Light, Etterzhausen, Germany) mounted on a 10 mm × 10 mm board area. In the center of this “blue COB” is a 6.5 mm diameter hole, through which fluorescence emitted from the surface of a sample can pass to the ST-detector contained in the ST-lamp. A second ST-detector is mounted at 90° angle relative to the ST-lamp in the Optical Unit (see below). While 20 of the total 22 LED chips serve for providing ST-flashes over a wide range of widths and intensities (see below), the remaining 2 chips serve for actinic illumination in applications where ST-kinetics are measured from the sample surface (e.g. with leaves). The “blue COB” also features two 1 mm holes for far-red (FR, 730–740 nm) illumination of the sample via so-called “pigtail LEDs” (plastic optical fibers coupled to LED-chips). A second FR-source is contained in the MCP-E II that is mounted diametrically to the ST-lamp in the Optical Unit (see below). The photodiode detector in the ST-lamp is protected by a red glass filter (RG665, Schott) in front of which a thin (0.1 mm) filter foil is mounted (Lee #135, deep golden amber) that absorbs most of the stray blue ST light, thus preventing excitation of red glass luminescence.

The 20 ST-LED chips are divided up into four chains in which each chain consists of 5 chips connected in series. The four chains are controlled by rapid current drivers placed close to the COB in order to keep the connecting wires short. Amplitude and width of the current pulses are controlled by the microprocessor via DA-conversion in combination with a rapid switch.

#### STK & PAM Detector unit (Combi-detector)

The STK & PAM Detector unit (Combi-detector) allows both PAM and STK measurements. It provides for a second PAM signal measured from the same sample under the same optical conditions in addition to the signal measured with the standard detector of the Multi-Color-PAM (MCP-D). Both detectors are equipped with the same pin-photodiodes and the same pulse amplifiers, so that practically equal responses are obtained as long as the same detector filters are used. By using different filters that pass more or less fluorescence originating from the pigment systems of PSII, F(II), or PSI, F(I), information on the distribution of excitation energy between the two photosystems can be obtained (Schreiber and Klughammer 2021). For this purpose, a combination of 2 mm RG665 + 1 mm RG9 (Schott) in front of MCP-D (F > 700, signal 1) and 2 mm RG665 + short-pass 710 (Balzers) in front of Combi-D (F < 710, signal 2) have proven appropriate.

In applications where differentiation between F(I) and F(II) is *not* intended, for optimal signal/noise ratio just 2 mm RG665 are placed in front of both detectors. In the case of STK measurements, however, depending on conditions, the Chl fluorescence measured by the Combi-D may be disturbed by a small RG665 luminescence signal that accumulates during the ST and decays after ST-off in the µs time range. The relative size of this optical artefact can be estimated with the help of a blank-sample (cuvette containing water). If judged too large, it can be completely avoided by placing a Lee #135 (Deep golden amber) filter foil in front of the RG665 filter.

Special care was taken for synchronization of the high speed STK-DA-conversion and the digital trigger pulse for the switch that controls the execution time and width of the STK-current pulses. In this way, an absolute jitter-free recording of the ST-kinetics was achieved (see Fig. 2). This is a prerequisite for the rising-edge correction approach described below (see section on ‘ST-profile correction’) which enables reaching sub-µs time resolution at the start of an ST and assessment of the initial fluorescence yield.

#### Multi-Color-Emitter unit

The optical and functional properties of the Multi-Color-Emitter (MCP-E II) have remained unchanged with respect to the original Multi-Color-PAM described in Schreiber et al. (2012). Regarding the electronics, however, the MCP-E II now incorporates the drivers for the various light qualities in order to avoid large current pulses through the detector cable and to improve the precision of the ML-pulses.

#### Experimental setup

**Fig. 1:**
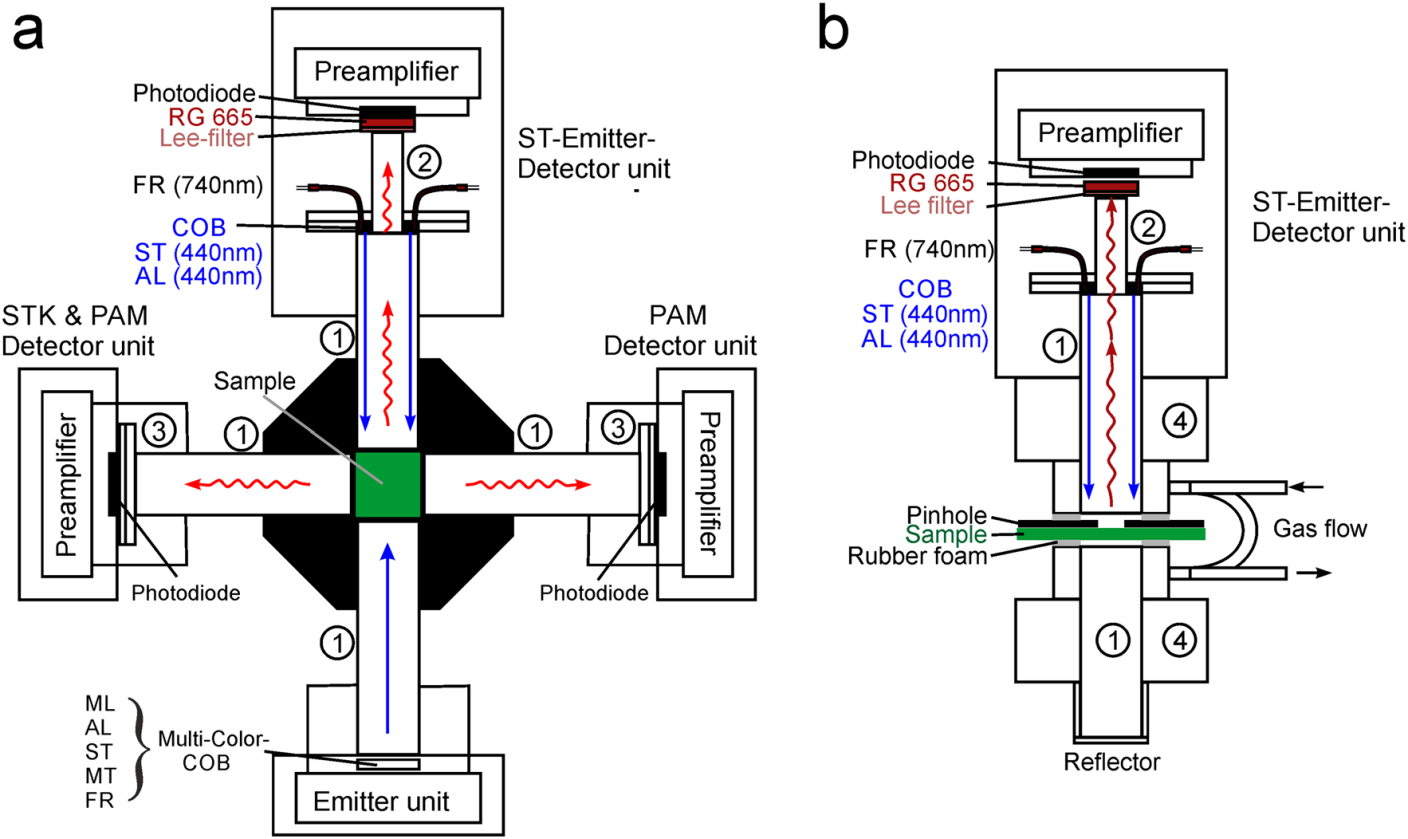
Block diagram of experimental set-up for combined measurements of PAM fluorescence and flash kinetics (STK) in suspensions (**a**) or from the sample surface (panel b). 10 × 10mm perspex light pipes (1) serve for guiding the various types of light to the samples and the fluorescence towards the detectors. At the heart of the measuring system is a new ST-Emitter-Detector unit (ST-lamp) featuring a powerful custom-made chip-on-board LED array (440nm COB), with a 6.5mm diameter central hole through which fluorescence reaches the photodiode detector via a 6.5mm perspex light guide (2). In **a** the 10 mm x 10mm suspension cuvette is located in the center of a standard PAM Optical Unit (ED-101US) with four optical ports, on which the Multi-Color Emitter unit, the ST-Emitter unit, the standard PAM Detector unit and the new STK & PAM Detector unit (Combi-detector) are mounted. The latter Detector units feature filter holders (3) for accomodating various filter sets. In **b** the ST-Emitter-Detector unit (ST-lamp) is mounted on a Leaf Measuring Head (3010) with gas flow connectors (4). For further details, see text

Figure 1 shows the experimental setup for combined measurements of PAM fluorescence and flash kinetics (STK) in a block diagram. In panels a and b the major opto-electronical components for measurements with suspensions and from the sample surface are depicted, respectively.

### Various types of light pulses available with the new device

The new instrument for combined measurements of PAM fluorescence and flash kinetics (STK) provides an unprecedented variety of light pulses, differing in intensity, width, pulse form, color and repetition mode, a detailed description of which would go beyond the scope of this communication. Some relevant information is presented under Supplementary Materials S1. The following main text will focus on the properties of ST flashes generated by the new ST-lamp.

### ST flash properties

#### Flash profile

**Fig. 2:**
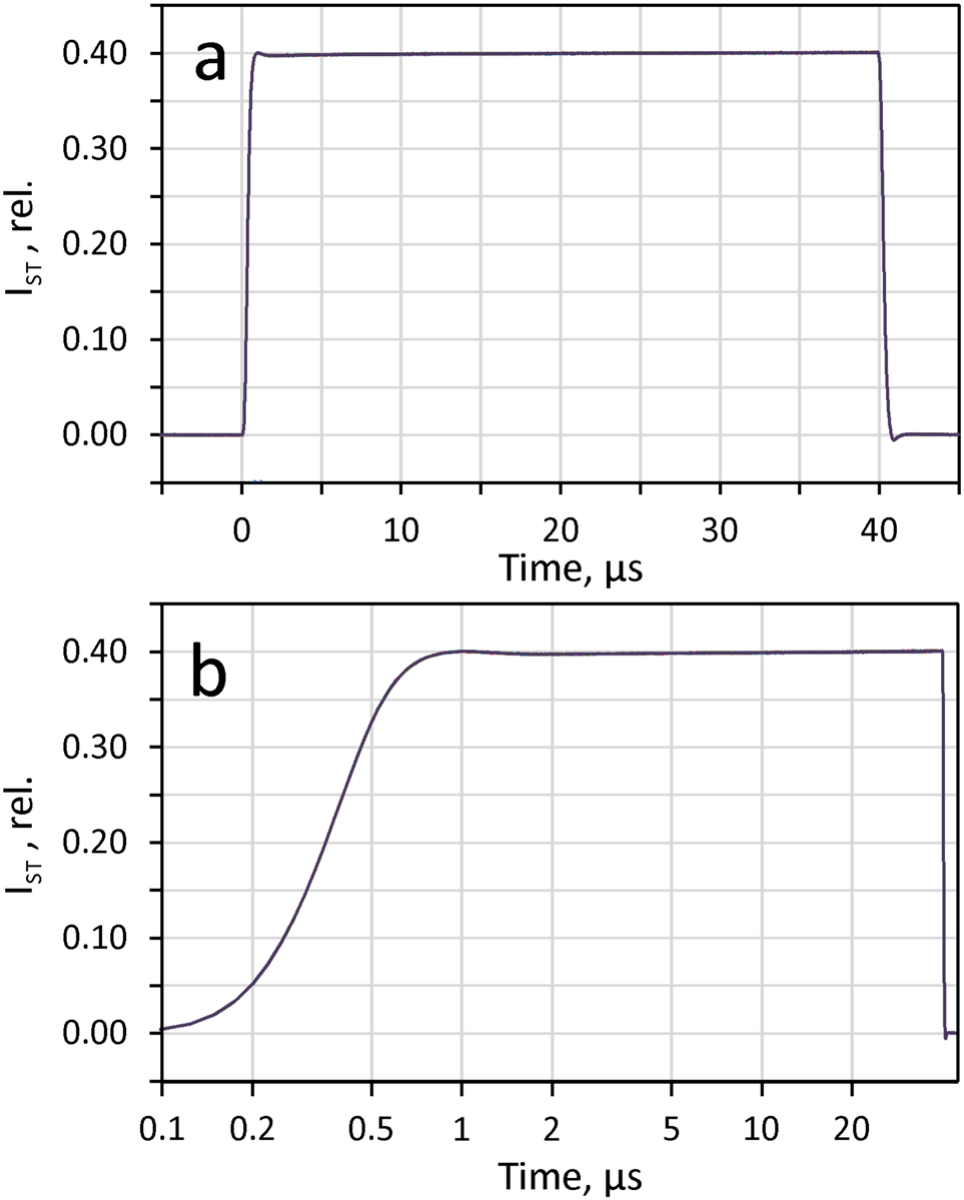
Profile of 40µs flash at 75% of maximal intensity: Superimposed responses of a sequence of 10 consecutive ST measured with the Combi-detector at 100ms repetition rate. **a** Linear time scale. **b** Logarithmic time scale. For details, see text

The electronic circuitry and microprocessor firmware was optimized for rapid rise and fall edges of the LED flash, a close to square profile, as well as maximal reproducibility of flash intensity and width. The detector and amplifier system was designed to measure the resulting ST-induced fluorescence signal with high accuracy. Figure 2 shows the profiles of ten 40 µs ST at 75% of maximal ST-intensity consecutively measured with 100 ms repetition rate using the Combi-D detector. As will be shown below (section on ‘Saturation of ST-induced variable fluorescence yield’), at this intensity even a much shorter 3 µs ST is saturating in experiments with dilute suspensions of *Chlorella*.The ST light reflected from a 45° surface mirror replacing the cuvette was measured. The intensity reaching the photodiode was attenuated with a 0.5% neutral density filter (Schott) in combination with a hole-diaphragm (5 mm hole in black-anodized aluminum plate). A 400 mV signal amplitude was fine-adjusted via the distance between the sliding filter holder and the photodiode (see Fig. 1, STK & PAM detector, feature 3).

Half-maximal intensity of the flash is reached in 0.3 µs and full intensity in 0.9 µs. The traces of the ten consecutively measured ST responses cannot be distinguished visually from each other, which shows that the signals are practically free of jitter and of fluctuations of width and intensity. The deviation of the relative intensity of an individual ST (I_ST_) from the mean is smaller than 10^–3^. Given the high reproducibility of the rising edge profile of the flash, the time resolution for assessment of the initial signals (amplitude of F_i_ orF_0_) can be considerably shifted below 0.9 µs, when the responses are corrected for the rising edge profile by appropriate reference measurements, in which glass and optical filter luminescence are avoided. This condition was fulfilled in the experiment of Fig. 2, by purposefully minimizing glass in the light path. As will be shown below, instead of using a 45° surface mirror, also the light scattered from a spherical quantum sensor placed in the center of the cuvette or simply the stray light reflected from a water filled cuvette may serve for reference measurement as well (see section below on ‘Slope and profile correction’).

#### Flash intensity

The intensity of the ST-flashes, in terms of the relative amplitude of the quantum flux density, depends on the supply voltage of the “blue COB” in the ST-lamp which can be set between 1000 and 3300 mV, resulting in a variation of quantum flux density by a factor of about 1000. As will be shown below (see section below on ‘Saturation of ST-induced fluorescence yield’), in a dilute suspension of *Chlorella* 95% closure of PSII is obtained with a 3 µs ST at about 30% of maximal ST-intensity. *Absolute* values of quantum flux density of photosynthetically active radiation (PAR) in units of 440 nm quanta/(m^2^s) can be estimated by comparison with the quantum flux density of a 440 nm MT applied with the 440 nm actinic LED array of the MCP-E. The latter intensity can be measured accurately with a spherical quantum sensor placed in the center of the illuminated water-filled part of the cuvette. Comparative intensity measurements of MT from the MCP-E and of ST from the ST-lamp can be carried out under close to equal optical conditions via the stray light that is scattered at right angle from the white sphere of the sensor towards the STK Detector (for optical geometry, see Fig. 1a).

The flash intensity applied in a particular experiment can be specified either in absolute units, like 440 nm quanta/(m^2^s), or in relative units, e.g. percent of maximal intensity. In practice, the relationship between ST-intensity and ST-lamp supply voltage is most relevant.The PamWin-4 program provides a routine for first averaging two 440 nm MT-pulse responses at maximal MT-intensity (MT20) and then measuring a list of ST-intensities as a function of 21 different custom supply voltages. The effective ST-intensities within the cuvette at the site of the sample are determined, based on the known maximal MT-intensity at the same site. Under Supplementary Materials S2 further details on flash intensity measurement and a list of custom ST-intensities determined with the applied instrument prototype is presented. With the given prototype, maximal ST-intensity (with 3300 mV supply voltage) amounts to 1.3 mol 440 nm quanta/(m^2^s). Theoretically, assuming linearity between photochemical rate and intensity, this flux density of blue quanta should suffice to saturate PSII charge separation in *Chlorella* within about 0.3 µs (Schreiber et al. 2012). In reality, however, the effective rate of charge separation is lower, as at high intensities a substantial part of the absorbed excitation energy is lost by non-photochemical quenching processes (Schreiber et al. 2019) (see sub-sections on ‘Lowering of F_m_^ST^ by HIQ ‘ and ‘Saturation of ST-induced fluorescence yield’).

The flash profile displays some dependence on the supply voltage, with the ST-on slope decreasing with decreasing voltage. In the case of short ST-pulses (< 5 µs), at supply voltages below about 1250 mV (i.e. below about 5% of maximal intensity), this causes a significant reduction of integrated flash energy, which may affect quantitative studies of fluorescence yield in the low ST-intensity range. In this case, the range of effective ST-intensities can be lowered by placing neutral density filters between cuvette and the perspex light guide that connects with the ST-lamp (e.g. a combination of non-fluorescing Lee #209 and Lee#211 filter foils, resulting in about 5% effective ST-intensity; total thickness 0.15 mm). An example of application is given in the section below on ‘Saturation of ST-induced fluorescence yield’.

#### ST-profile correction

**Fig. 3:**
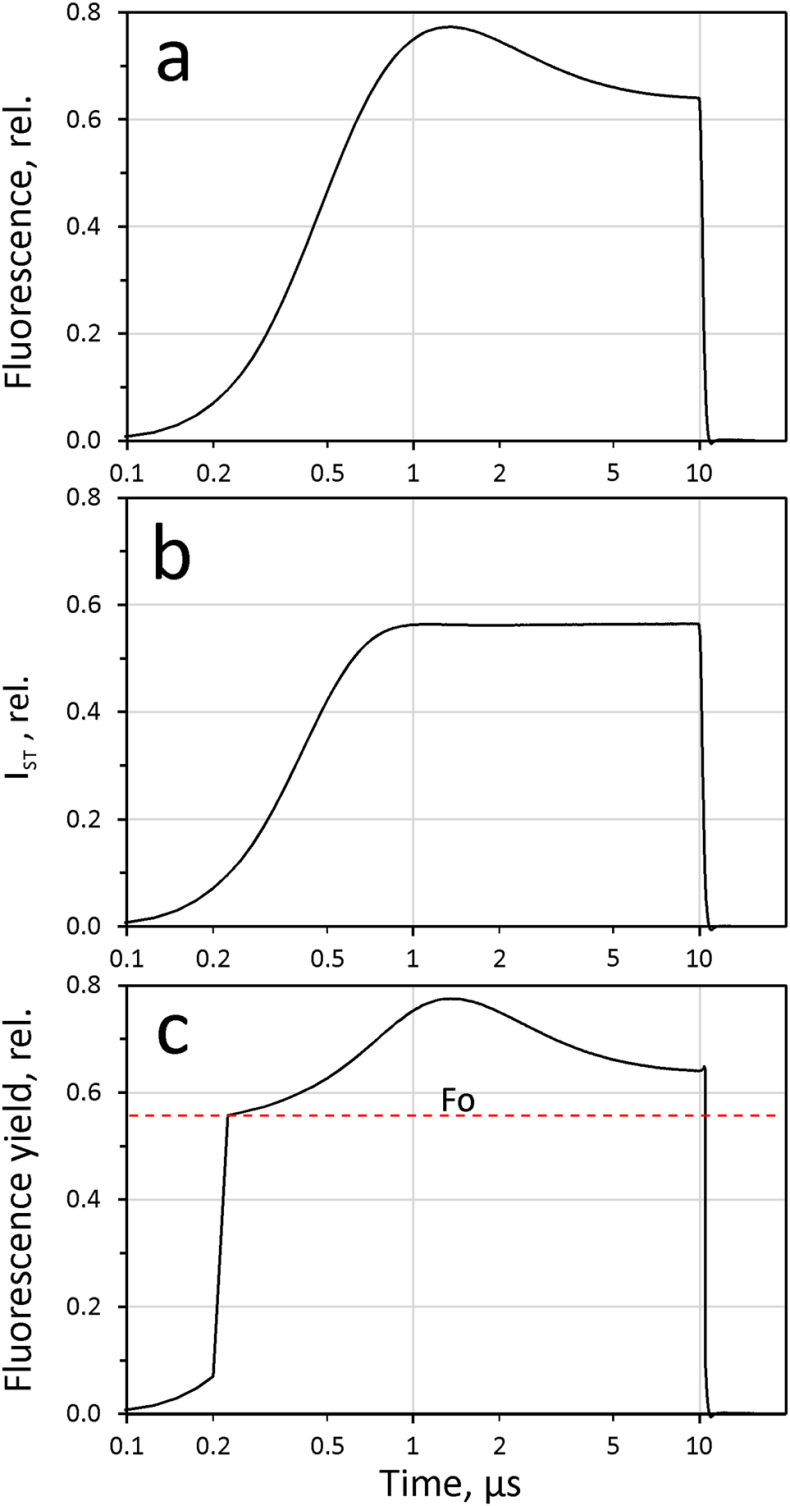
Example of ST rising edge correction. **a** Dilute suspension of Chlorella; original, uncorrected ST-kinetics (STK) measured in response to a 10µs ST at maximal ST-intensity (3300 mV supply voltage). **b** ST-profile measured with blank sample (unfiltered stray light) under identical instrument settings (reference measurement). **c** Chlorella STK of panel a corrected with ‘Reference’ response shown in panel b. Correction threshold, 15%. Logarithmic time scale

In view of the rectangular ST-profile depicted in Fig. 2, in many applications no correction is required, as it may be assumed that the intensity is practically constant from 1 µs after triggering to the end of the ST. Using standard ST-intensities that assure full closure of RCII within about 40 µs, no essential information is apparent in the sub-µs time range. In special applications, however, e.g. in the study of high-intensity quenching (HIQ), availability of much higher ST-intensities are of interest, where Q_A_-controlled photochemical fluorescence quenching already decreases in the sub-µs range, in competition with carotenoid triplet quenching (TQ, a major component of HIQ) and potential other forms of HIQ. In this case, time resolution can be considerably improved by ‘rising edge” or ‘ST-profile’ correction. An example is given in Fig. 3.

In the original *Chlorella* ST-kinetics recording at maximal ST-intensity in Fig. 3a the fluorescence increase due to closure of PSII reaction centers (RCII) cannot be distinguished from the rising edge of the ST. The latter corresponds to an ‘instrument function’, the course of which is displayed in panel b, recorded by a separate ‘Reference’ measurement. The latter monitors the stray light that is scattered by a blank sample under identical instrument settings. ST-profile correction involves division of the original data by this ‘Reference’ response. The result is shown in panel c. The PamWin-4 program allows to define a threshold down to which the ‘Reference’ signal shall be applied for correction. In the given example this threshold amounted to 15%. In this case, assessment of initial fluorescence, F_0_, was at 0.3 µs.

### Twin flashes for measuring dark relaxation, assessment of HIQ and determination of F_m_^ST^

The changes of fluorescence yield measured at maximal flash intensity that are displayed in Fig. 3c are composed of a light driven increase, reflecting reduction of the primary PSII acceptor Q_A_, and the light driven formation of strong fluorescence quenching. For the latter we have introduced the collective term ‘HIQ’ (Schreiber et al. 2019), which besides carotenoid triplet quenching (TQ) may include quenching by P680^+^ and possibly other forms of ‘donor-side dependent quenching’(DQ). The new measuring system enables to differentiate between the increase of fluorescence yield by Q_A_ reduction and its suppression via HIQ by application of a second ST (ST#2) after a defined period of dark-time (‘twin STK’ recording). HIQ relaxes in the dark following ST#1-off. As far as TQ is concerned, relaxation should be largely completed within 20 µs, i.e. at a time where Q_A_^−^ reoxidation may be considered to be negligibly low. P680^+^ reduction should be even faster, except for a small fraction in equilibrium with its oxidized primary donor, Yz(ox) (or Yz^+^) (for a recent review, see Shevela et al. 2023). Hence, when ST#2 is applied 20 µs after a saturating ST#1, it may be expected that the *initial fluorescence yield (F*_*i*_*) measured in ST#2* does reflect a particular state with 100% Q_A_^−^ in the absence of TQ, which may be referred to as ‘relaxedF_m_^ST^’. However, while ‘relaxed F_m_^ST^’ should be almost free of TQ, it is still modulated by slowly relaxing forms of DQ, as will be demonstrated below (see section on ‘Sequences of ST-kinetics of period-4 oscillations’) and, hence, it is lower than the maximal fluorescence yield F_m_ measured by PAM fluorimetry using an MT-protocol which not only assures relaxation of TQ, but of DQ as well.

**Fig. 4:**
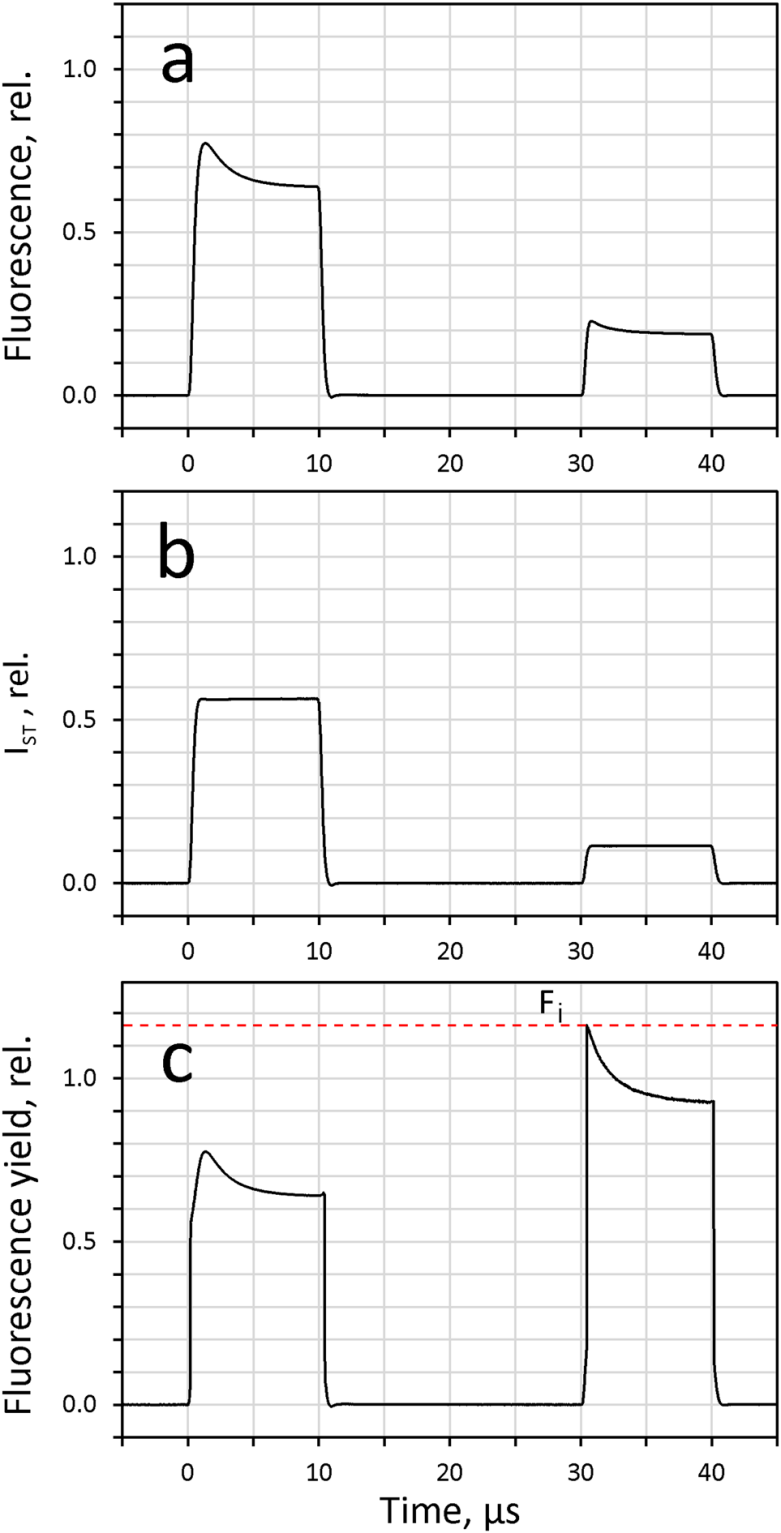
Example of twin ST-profile correction of the data presented in Fig. 3. **a** Dilute suspension of Chlorella; original, uncorrected ST-kinetics (STK) measured in response to twin 10µs ST (ST#1, maximal intensity, ST#2, 20% of maximal intensity). 20µs dark time between ST#1 and ST#2. **b** Twin ST-profile measured with blank sample (unfiltered stray light) under identical instrument settings (Reference measurement). **c** Chlorella twin STK of panel a corrected for twin ST-profile shown in **b**. Correction threshold, 15%. Linear time scale

At high ST-intensity, upon onset of ST#2, TQ formation is very rapid, so that accurate determination of F_i_ is a challenging task. In practice, this problem can be solved by attenuating the intensity of ST#2 with respect to that of the ST#1, followed by appropriate correction of the ‘twin ST’ fluorescence changes based on the ‘twin ST’ intensity profile. Using this technique, the relaxed F_m_^ST^ can be determined even at maximal ST-intensity. An example is presented in Fig. 4 using the same sample of *Chlorella* and the same maximal intensity of ST#1 as in the measurements of Fig. 3. Differently from Fig. 3, the data are presented on a linear time scale.

The twin ST technique demonstrated in Fig. 4 enables a thorough investigation of the reactions induced by flashes of strong and supersaturating light. In particular, this technique may open the way for a better understanding of various types of non-photochemical quenching in vivo that lower the fluorescence yield measured with an ST-protocol compared to the MT-protocol applied in PAM fluorimetry (saturation pulse quenching analysis).

### Sequences of STK (STKS) and interval analysis

Sequences of saturating ST (or flash trains) have been playing an important role in photosynthesis research, since Joliot et al. (1969) first reported on period-4 oscillations in the yield of oxygen evolved by individual ST, when isolated chloroplasts or *Chlorella* were illuminated by a series of saturating 10 µs ST separated by 300 ms dark-times. The new device displays exceptional flexibility in programming ST over a wide range of intensities, widths, numbers of ST and repetition times (i.e. dark-intervals between consecutive ST). These setting parameters are automatically saved together with every STK-recording, so that they can be readily re-installed for reproduction of measurements with a particular set of settings.

**Fig. 5:**
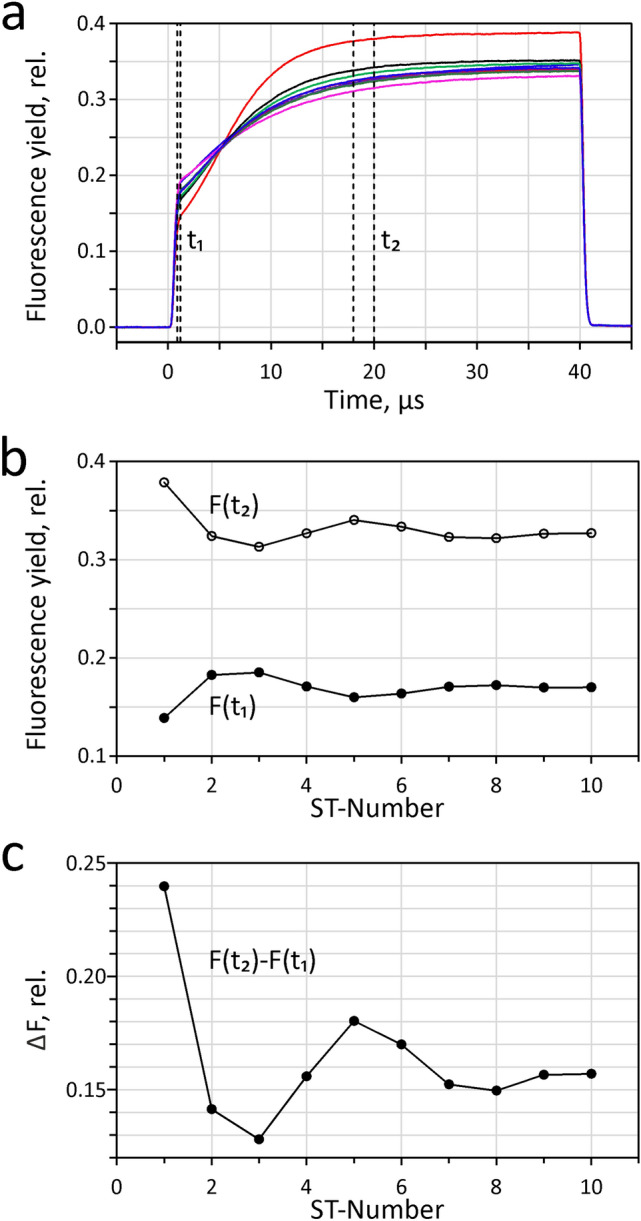
STKS measurement with a dilute suspension of dark-adapted Chlorella.Ten consecutive 40µs ST at 5% of maximal ST intensity applied with 100ms repetition time. **a** The superimposed STK traces, with vertical lines defining time intervals t_1_ = 0.9–1.2µs and t_2_ = 18-20µs for determination of F_0_ and F^ST^, respectively. **b** Plots of the F_0_-values, i.e. F(t_1_), and the F^ST^-values, i.e. F(t_2_), determined by averaging the data points in the defined intervals. Panel c, plot of the difference between F^ST^ and F_0_, i.e. F(t_2_)-F(t_1_)

In Fig. 5 an example of a typical measurement of an STK sequence (STKS) with dark-adapted *Chlorella* is presented: 10 consecutive ST were applied at 5% of maximal ST intensity, 40 µs width and 100 ms repetition time. Under these conditions pronounced period-4 oscillations of F_0_ and F_m_^ST^ are observed which reflect the 4-step cooperation of positve charges in the oxygen evolving complex (OEC) at the donor-side of PSII (Kok et al. 1970; Delosme 1971a, b) (see section below on ‘Sequences of ST-kinetics and period-4 oscillations: Delosme (1971) revisited’). The PamWin-4 software provides a dedicated routine for plotting and analysing the complex information contained in an STKS recording. Figure 5 shows three different plots after export to Excel. In panel a the superimposed STK are depicted. Notably, not only the amplitudes of the consecutive responses, but also their kinetics display remarkable differences. The STK#1 (red curve) stands out by showing the lowest F_0_ and highestF_m_^ST^. All curves show a common crossing point at 5.3 µs. Two time intervals (black vertical lines) were defined for analysis of the changes in fluorescence yield that are contained in the various STK at t_1_ = 0.9–1.5, for assessment of F_0_, and t_2_ = 18-20 µs, for F^ST^. In panel b, the averaged data points of the F_0_ and F^ST^ intervals are plotted against the ST-number. The difference between F^ST^ and F_0_, F(t_2_)-F(t_1_), is plotted in panel c.

It may be noted that the STK depicted in panel a are *single responses*, i.e. obtained *without averaging*, and still are practically free of noise. This aspect is important for reliable assessment of fluorescence properties in particular physiological states in vivo, which depend on numerous external and internal parameters and rarely can be kept constant over a longer period of time that would be required for meaningful averaging.

## Results and interpretation

The new measuring system is outstanding in that it enables to carry out *comparative measurements* of pulse-modulated fluorescence yield (PAM) and ST-kinetics (STK) *on the same sample* in a given physiological state. While in the past, both of these two measuring techniques have led to important new insights, some important questions concerning the relationship between fluorescence and photosynthesis have remained unanswered. In particular, considerable uncertainties have arisen from the fact that maximal fluorescence yield induced by a saturating single turnover flash, F_m_^ST^ (ST-protocol) is consistently lower than the F_m_ measured by PAM fluorimetry and the Saturation Pulse routine (SP quenching analysis, MT-protocol). The nature of fluorescence quenching that lowers F_m_^ST^with respect to F_m_ has been intensively studied and debated during the past decades, so far without reaching a satisfactory general consensus. In these previous studies, vastly different types of samples were used, reaching from PSII particles to intact leaves, thus complicating a fair comparison of previous results, in addition to differences caused by the use of various types of measuring techniques. All measurements of the present study were carried out using dilute suspensions of *Chlorella *in vivo under conditions allowing a quantitative comparison of the fluorescence yields measured by application of pulse-modulated light and flash illumination.

### *Quantitative comparison of the relative fluorescence yields measured *via* PAM fluorimetry and ST-kinetics (STK)*

**Fig. 6:**
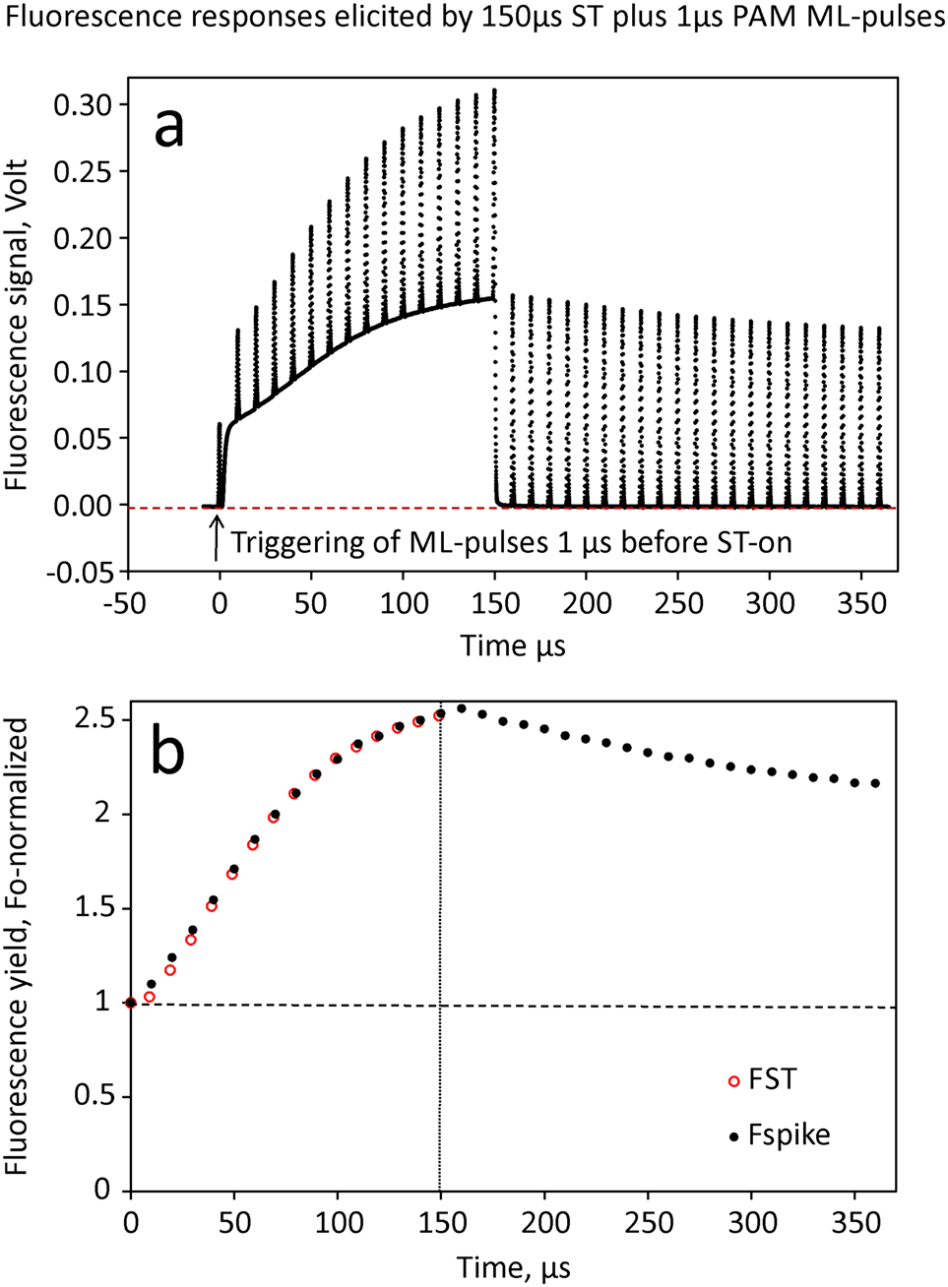
Quantitative comparison of fluorescence signals elicited by 150µsST and 1µs PAM-ML pulses in a dilute suspension of Chlorella. The intensity of the ST was adjusted to the same value as that of the PAM-ML pulses. **a** Original recording of the ST-induced changes of fluorescence signal, with the PAM ML-pulses (at 100 kHz) being triggered 1µs before onset of the ST at time = 0. **b** F_0_-normalized fluorescence excited by the PAM ML-pulses (black points), compared with F_0_-normalized fluorescence excited during the course of the ST (red circles)

For quantitative comparison of fluorescence yields, it is important that both types of signals can be measured in the same sample under equal conditions using the same detector. As shown in Fig. 1a above (experimental set-up for combined measurements of PAM fluorescence and flash kinetics), Chl fluorescence is equally excited by the PAM ML-pulses originating from the Multi-Color-Emitter unit and by the flashes originating from the ST-lamp. The fluorescence is equally guided towards the STK & PAM Detector unit. The STK detector equally responds to the fluorescence excited by a flash from the ST-lamp and by PAM ML-pulses. For a demonstration of the equivalence of both responses, the intensities of ST and PAM ML pulses can be equalized and the pulse-modulated ML triggered 1 µs before onset of the ST (as outlined under Supplementary Materials S3). The result is shown in Fig. 6.

In panel a the original combined responses of fluorescence excited by the 100 kHz PAM ML-pulses and the 150 µs ST are displayed. The changes in fluorescence yield are almost exclusively driven by the continuous light of the ST. At the given intensity, the 1 µs PAM ML-pulses are too short to induce significant changes in fluorescence yield during the 150 µs illumination period. They do, however, affect the relaxation kinetics after ST-off at 150 µs (not shown in the figures). In panel b the amplitudes of the PAM ML-pulse excited responses (spikes) are compared with the corresponding amplitudes of the ST excited fluorescence, both normalized at F_0_. The two responses are close to equal, suggesting that the fluorescence yield associated with a given state of PSII in principle is equally well assessed by both approaches. Consequently, when differences are observed, as is the case when maximal fluorescence yield is determined by MT (via PAM) compared with ST protocols, these must be caused by differences in the physiological states induced by saturating MT and ST.

### Comparison of ST-kinetics (STK) and MT-induced polyphasic rise kinetics measured with the same dilute suspension of Chlorella

Dark–light fluorescence induction responses in vivo strongly depend on the physiological state of the sample. For quantitative comparison of ST- and MT-induced responses, particular attention has to be paid to the prehistory of illumination and dark acclimation. There is not only a continuum of different light states, but of different dark states as well. In algae the latter is controlled by the rate with which stroma reductants feed electrons into the plastoquinone (PQ)-pool (chlororespiratory flux), the redox state of which regulates reversible state 1–2 transitions, the ratio of Q_B_^−^/Q_B_ and the S-state distribution of the oxygen evolving complex (OEC). Suspensions of *Chlorella* display a stable and reproducible state at predawn, when the dark rate of PQ reduction is low and the enzymatic reactions downstream PSI are thoroughly dark-inactivated.

**Fig. 7:**
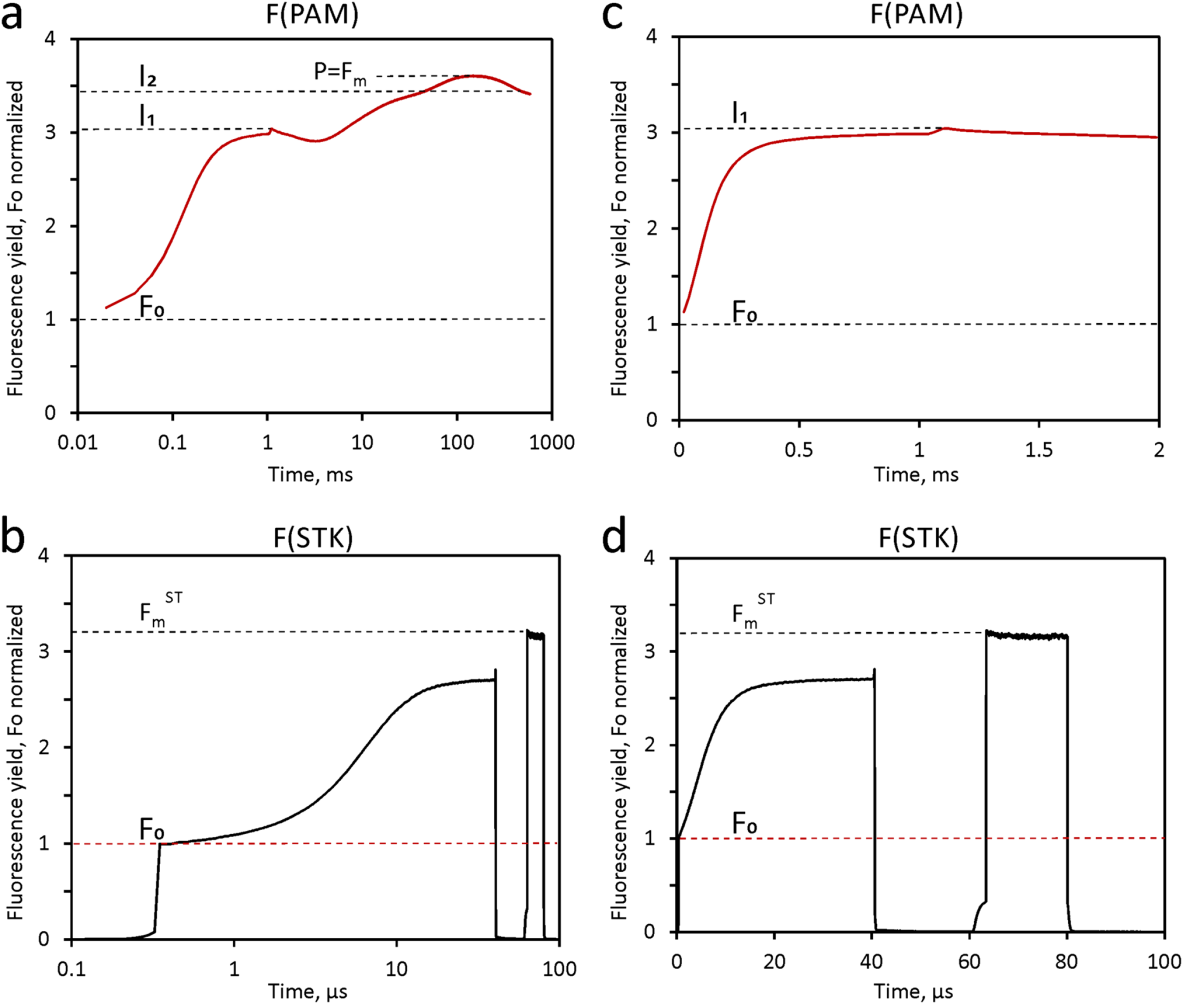
Comparative PAM and STK measurements with the same dilute suspension of dark-adapted predawn Chlorella. F(PAM), polyphasic fluorescence rise induced by 600ms MT at 3550 µmol 440nm quanta/(m^2^s). At 1 ms a saturating 50µs Multi-Color-ST is applied for complete reduction of Q_A_ and determination of the I_1_ level. F(STK), twin ST (40µs ST#1 at 5% of maximal ST intensity, amounting to 65,000 µmol 440nm quanta/(m^2^s), followed by a 10 × weaker 20µs ST#2 after 20µs dark-time. First the STK and 5min later the polyphasic rise kinetics were measured. Data normalized at initial fluorescence yields, F_0_. F(STK), twin ST-profile corrected. **a** and **b** complete responses using logarithmic time scale. Panels c and d, responses corresponding to the O-I_1_ transient and the increase from F_0_ to F_m_^ST^, respectively

In the experiment of Fig. 7 predawn samples of a dilute suspension of *Chlorella* are used for comparison of MT- and ST-induced responses. The STK were corrected using the twin ST-profile technique outlined above (see Fig. 4). Figure 7 shows the data after export to Excel and normalization of the initial fluorescence yields, F_0_. While the PAM recording shows the polyphasic rise kinetics induced by a 600 ms MT at 3550 µmol 440 nm quanta/(m^2^s), the STK recording displays the response to a twin ST (40 µs ST#1 at 5% of maximal ST intensity, amounting to 65,000 µmol 440 nm quanta/(m^2^s), followed after 20 µs dark-time by a 10 × weaker 20 µs ST#2). In Fig. 7 a, b the complete responses are compared using a logarithmic time scale. The rapid responses corresponding to the O-I_1_ transient and the fluorescence increase from F_0_ to F_m_^ST^ are compared using linear time scales in panels c and d, respectively.

By comparison of the normalized PAM and STK responses of dark-adapted predawn *Chlorella* four major findings are apparent:

1. The fluorescence yields reached at the I_1_-level (PAM) and F_m_^ST^ (STK) are similar, but not equal. I_1_ amounts to 3.0 and F_m_^ST^ to 3.2 F_0_ units. The latter applies for the fluorescence yield assessed by ST#2, i.e. *after relaxation of HIQ* and *not* for the fluorescence yield reached *during* ST#1, which is appreciably suppressed by HIQ.
2. Both I_1_ and F_m_^ST^ are substantially lowered with respect to the peak F(PAM) yield (P or Fm), which is observed at about 160 ms in the MT-induced polyphasic kinetics and amounts to 3.6 F_0_ units.
3. With the I_2_-level (PAM) at 3.35 F_0_ units, the differences to I_1_ and F_m_^ST^ are relatively small, amounting to 0.35 and 0.15 F_0_ units. Hence, considering that I_2_ reflects maximal PSII fluorescence, F_m_(II) (Schreiber and Klughammer 2021; Schreiber 2023, see also following section on simultaneous F > 700 and F < 710 measurements), the gap between the F_m_(II) determined by ST- and MT-protocols is distinctly smaller than concluded from previous studies (Samson and Bruce 1996; for a recent review, see Garab et al. 2023).
4. In spite of the vastly different intensities of the applied MT and ST (factor of 18.6), the initial sigmoidal rise kinetics are quite similar. The half-time, t_1/2_, of the MT-induced rise is 120 µs. If PSII turnover would follow the product of pulse intensity and pulse width (Ixt), the t_1/2_ of the ST-induced rise should be 120/18.6 = 6.5 µs. In reality, it formally amounts to 5.5 µs, based on the terminal value at 40 µs. However, it has to be considered that the fluorescence rise caused by Q_A_ reduction is superimposed by a decline due to HIQ development. When t_1/2_ determination is based on the fluorescence yield after HIQ relaxation (F_i_ in ST#2), it amounts to 7.2 µs.

The responses presented in Fig. 7 were obtained from measurements with a thoroughly dark-adapted sample of predawn *Chlorella,* explicitly avoiding any kind of preillumination by ambient background light (see Materials and Methods)*.* In cases when the sample inadvertently was preilluminated by weak ambient light, the responses tended to show somewhat higher values of F_0_ and considerably lower values of I_1_ and F_m_^ST^.

**Fig. 8:**
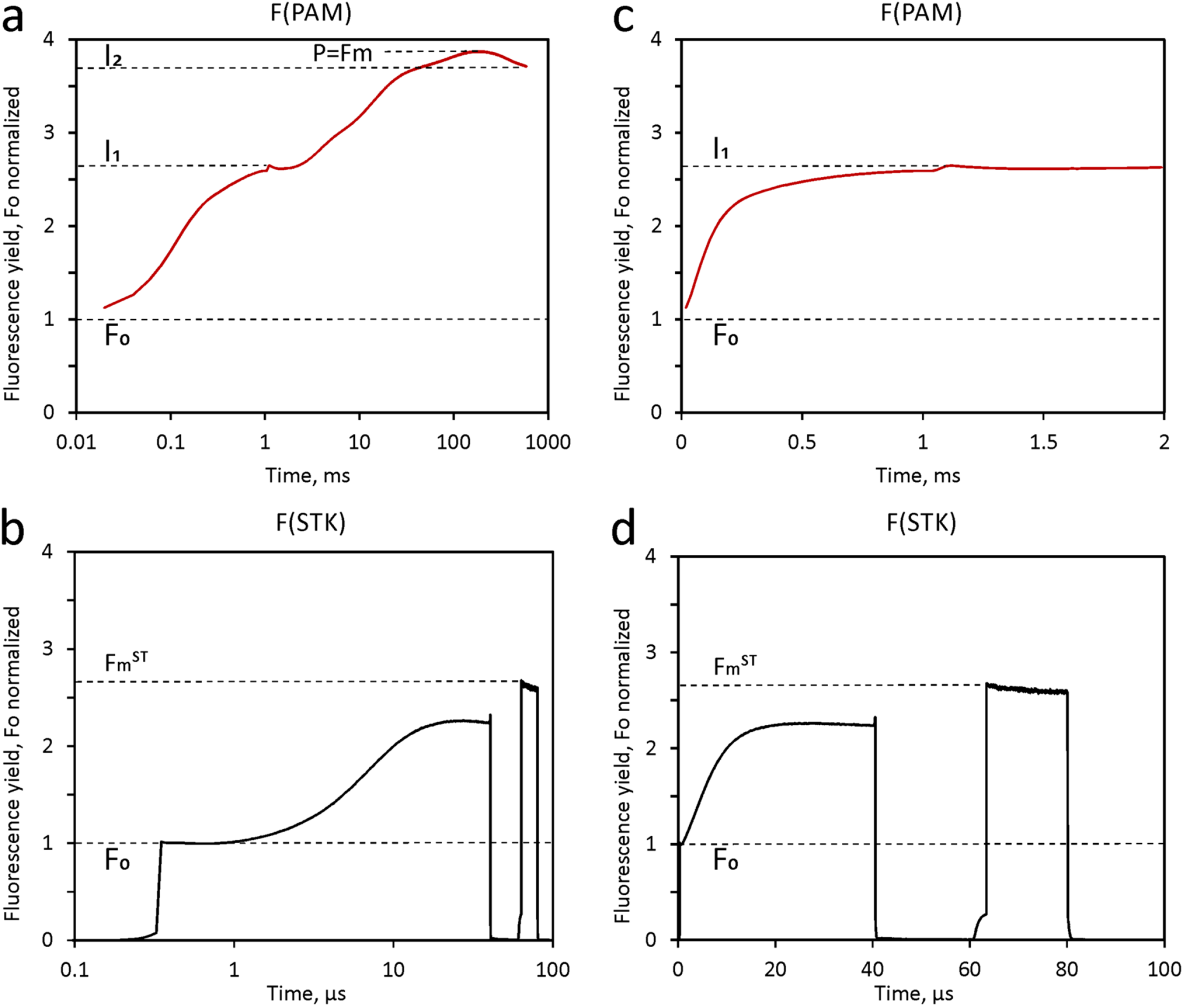
Comparative PAM and STK measurements with the same dilute suspension of predawn Chlorella exposed to weak FR background light. PAM, polyphasic fluorescence rise induced by 600ms MT at 3550µmol 440nm quanta/(m^2^s). STK, twin ST (40µs ST#1 at 5% of maximal ST intensity, amounting to 65,000 µmol 440nm quanta/(m^2^s), followed by a 10 × weaker 20µs ST#2 after 20µs dark-time). Data normalized at initial fluorescence yields, F_0_. STK, twin ST-profile corrected. Same conditions as in measurements of Fig. 7, except for the FR background illumination, the intensity of which amounted to 2 µmol 730–740 nm quanta/(m^2^s), corresponding to about 1/300 of maximal FR intensity provided by the Multi-Color Emitter unit

Analogous measurements were carried out under *controlled conditions of preillumination* with a predawn *Chlorella* sample that after transfer to the cuvette was continuously illuminated with weak far-red light (FR) at 2 µmol 730–740 nm quanta/(m^2^s) (Fig. 8) and for comparison also with weak 540 nm light (see Supplementary Materials S6). Separate PAM measurements allowed to estimate the rate of charge separation in PSII driven by such low FR quantum flux density from the kinetics of the fluorescence rise that is induced by maximal (about 300 times higher) FR-intensity in the presence of DCMU. While the estimated rate of one Q_A_ turnover every 40 s appears rather low to cause a significant effect via PSII activity, its overall effect on both the MT- and ST-induced responses is remarkable. In particular, I_1_ and F_m_^ST^ are substantially suppressed. I_1_ is lowered from 3.0 to 2.6 F_0_ units and F_m_^ST^ from 3.2 to 2.7 F_0_ units. At the same time, I_2_ is increased from 3.35 to 3.65, so that the amplitude of the I_1_-I_2_ rise is increased by a factor of 3. In contrast, the amplitude of the O-I_1_ transient is decreased from 2 to 1.5 F_0_ units.

The amplitude of the I_1_ (or J) level is known to increase with reduction of the PQ-pool between the two photosystems (Schansker and Strasser 2005; Toth et al. 2007), which argues for the observed FR-effect to be due to PSI activity, affecting PSII via the redox state of PQ and/or the secondary PSII acceptor Q_B_. In the case of F_m_^ST^ lowering by weak FR, however, a direct effect on PSII cannot be excluded. As will be shown below (sections on **‘**S-state advancement induced by weak FR in *Chlorella’*and ‘S-state advancement induced by weak FR in a dandelion leaf’) weak FR background light causes a forward shift of the S-states of the water-splitting enzyme system (OEC). In principle, this could be explained, if under in vivo conditions the predawn dark state would be characterized by a substantial amount of S_0_, which via PSII turnover could be slowly converted into S_1_ even by weak FR. This interpretation, however, is questioned by preliminary data presented under Supplementary Materials S6.

### Simultaneous measurements of F > 700 and F < 710: Assessment of F_v_(I) component

The polyphasic fluorescence rise induced by a multiple-turnover saturating pulse of light consists of ‘photochemical’ and ‘thermal’ components (Delosme 1967). While the rate of the photochemical phase (O-I_1_) is determined by the rate of PSII charge separation, the thermal phase is limited by dark-reactions. The latter is divided into two sub-phases, I_1_-I_2_, and I_2_-P (Schreiber 1986; Neubauer and Schreiber 1987). Recent work has revealed that fluorescence excitation and emission properties of I_2_-P differ significantly from those of the preceding O-I_1_ and I_1_-I_2_ phases, consistent with the suggestion that I_2_-P reflects variable fluorescence of PSI, F_**v**_ (I) (Schreiber and Klughammer 2021; Schreiber 2023).

The I_2_-P transient is more pronounced when fluorescence is measured at wavelengths longer than700nm (F > 700), compared with fluorescence measured at wavelengths shorter than 710 nm (F < 710). For quantitative comparison a special normalization routine of the light-induced responses (polyphasic rise kinetics O-I_1_-I_2_-P-S) has proven useful. Relying on the basic assumption that in both responses the O-I_1_ rise is due to F_v_ (II), after equalizing the O-I_1_ amplitudes of the two responses, all F_v_ (II) components should be equal and the difference should reflect F_v_ (I).

**Fig. 9:**
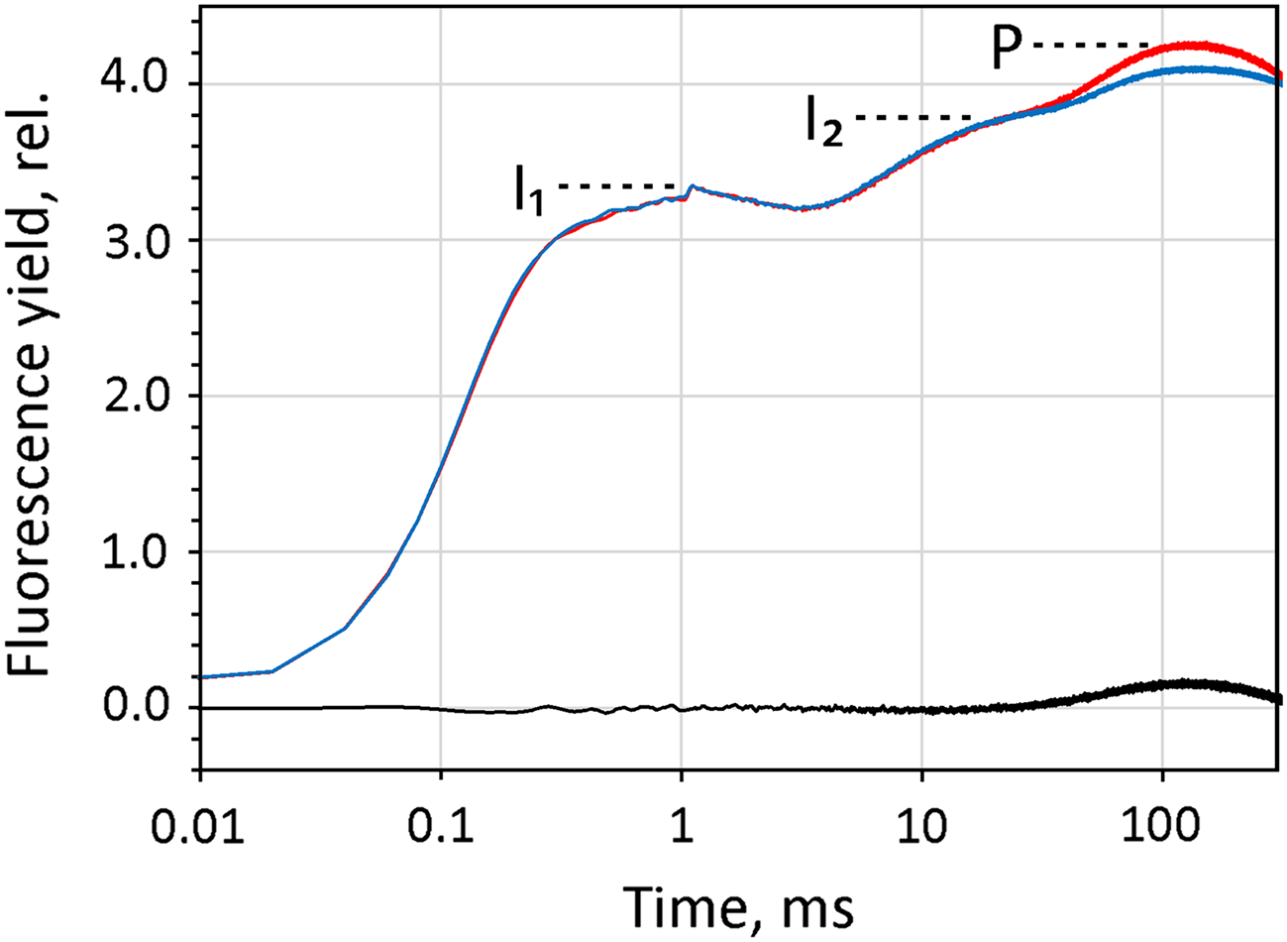
Simultaneously measured polyphasic rise kinetics of F_v_ > 700 (red) and F_v_ < 710 (blue) of dark-adapted predawn Chlorella. O-I_1_ normalized responses. The difference curve (black) reflects the kinetics of F_v_(I)

In the preceding work using the original version of the Multi-Color-PAM fluorimeter, measurements of F > 700 and F < 710 were carried out alternatingly, using the same detector and changing the detector filters. In this case, particular attention had to be paid to assuring that measurements of F > 700 and F < 710 were carried out under as close as possible equal physiological conditions, which is cumbersome. The new version of the instrument provides two equal fluorescence detectors (in optically equal positions) that can be equipped with different filter sets. Hence, comparative measurements of F > 700 and F < 710 have become more easy and more reliable. An example is given in Fig. 9 which shows simultaneously measured F > 700 (red) and F < 710 (bue) recordings of the polyphasic rise kinetics of predawn *Chlorella*. A dedicated O-I_1_ normalization routine provided by the PamWin-4 program was applied. While the two recordings are close to equal up to the I_2_-level, they differ selectively during the I_2_-P-S transient, which is more pronounced in F > 700. The difference between F_v_ > 700 and F_v_ < 710 reflects F_v_ (I) (black trace), which is induced when both acceptor and donor sides of PSI are reduced (Schreiber and Klughammer 2021). In the evaluation of the F_v_(I) amplitude, it has to be considered that also F < 710 contains F(I), so that the F_v_(I) revealed by the difference curve reflects less than the total F_v_(I) contained in F_v_ > 700. This aspect is particularly relevant in view of recent evidence suggesting that LHCII is a constitutive part of the PSI antenna system in vivo (Galka et al. 2012, Wientjes et al. 2013, Grieco et al. 2015, Chukhutsina et al. 2020). With this relatively new information in mind, it appears likely that in both responses all of I_2_-P-S is due to the transient appearance of F_v_(I) during dark–light induction (Schreiber 2023).

Identification of I_2_-P = F_v_(I) bears on the long-standing discussion on the mechanisms responsible for the difference in maximal fluorescence yields obtained by ST- and MT-protocols, F_m_^ST^ and F_m_ (or P), respectively. As was shown in Figs. 7, 8 above, a major part of this difference (corresponding to I_1_-I_2_) is controlled by the PQ redox state. Details on the mechanism by which oxidized PQ lowers fluorescence yield remain to be elucidated. With the rest of the difference (corresponding to I_2_-P) now being considered to be due to F_v_(I), this part does not need to be taken into account anymore, when the discrepancy between ST- and MT-induced changes of fluorescence yield is discussed.

### Sequences of ST-kinetics and period-4 oscillations: Delosme (1971) revisited

As already outlined under Materials and Methods (section on ‘Sequences of STK (STKS) and Interval Analysis’), the new device not only allows to assess the changes of fluorescence yield that are induced by a series of consecutive ST, but also to record *the kinetics of these changes* and to analyse them in detail. Furthermore, as described under Materials and Methods (section on ‘ST-profile correction’), it is possible to evaluate the distortion of these changes by high energy quenching (HIQ, Schreiber et al. 2019) after appropriate ‘ST-profile correction’.

The foundation for the results presented here was laid more than 50 years ago by the pioneering work of René Delosme, who for the first time succeeded in measuring the fluorescence yield *during* a saturating flash, i.e. in a state in which the primary PSII acceptor Q_A_ is fully reduced (Delosme 1971a, b). He discovered similar period-4 oscillations in the maximal fluorescence yield, F_m_^ST^, induced by a series of saturating single turnover flashes, as previously observed by Joliot et al. (1969, 1971) in oxygen evolution and in the initial fluorescence yield. After application of a series of consecutive flashes, separated by 1 s dark intervals, he found maxima of F_m_^ST^ for ST numbers 1, 5, 9 and 13 and minima for numbers 3, 7 and 11, the latter correlating with the sum of S_2_ + S_3_, which was known to correlate with the yield of ST-induced oxygen evolution (Joliot et al. 1971). Based on this finding, he concluded that the variation of quenching at the F_m_^ST^ level is caused by variation of the number of positive charges stored in the oxygen evolving complex of the water-splitting system (OEC). Later work by various researchers (reviewed in Delosme and Joliot 2002) has lead to the current understanding that the oxidized primary PSII donor, P680^+^, in equilibrium with the oxidized secondary donor molecule of tyrosine Z, Yz(ox) (or Yz^+^), is the actual fluorescence quencher. With an increasing number of positive charges stored in the OEC, this equilibrium is shifted towards the quencher P680^+^.

**Fig. 10:**
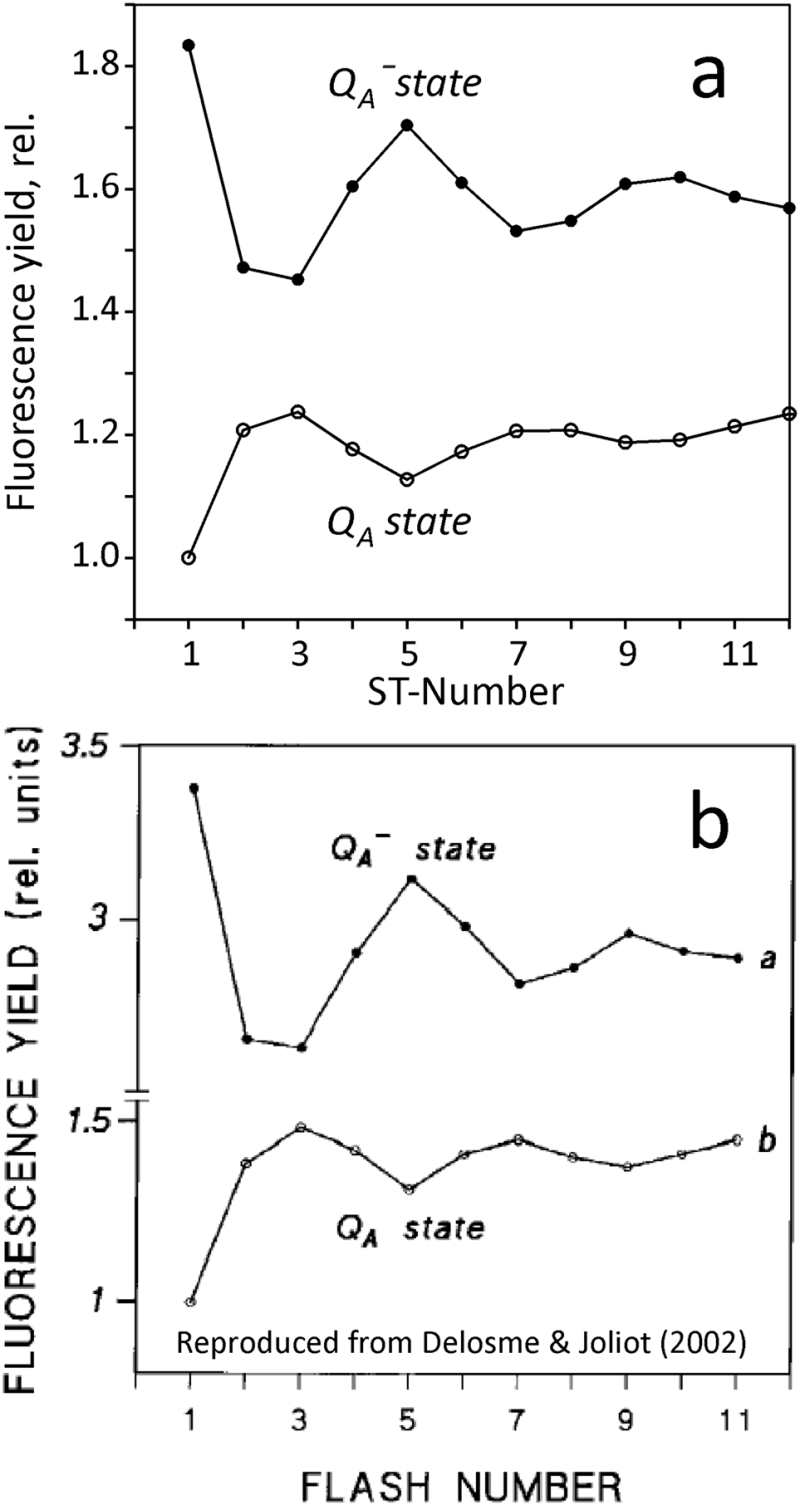
Fluorescence yield measured in a sequence of saturating 2µs flashes in Chlorella: Top curves, Q_A_^−^ state, maximal yield F_m_^ST^ as a function of flash number. Bottom curves: Q_A_ state, minimal yield F_0_ as a function of flash number. Panel a, measurement by the authors using the new device; 10°C, 100ms repetition time, 0.4 µmol 730–740 nm quanta/(m^2^s) background light. Panel b, reproduced from Delosme and Joliot (2002); temperature 0°; repetition time, 1s

We have tried to reproduce the data of Delosme (1971a, b) with our new device, measuring a sequence of ST-kinetics (STKS) with a dilute suspension of dark-adapted *Chlorella*. Similarly as in the original work, the applied ST caused complete closure of PSII within about 1 µs and F_m_^ST^ was assessed *during* the ST. Differently from the original work (Delosme 1971a, b), temperature was 10 °C (instead of 0 °C) and flash repetition time was 100 ms (instead of 1 s). For optimal correspondence between the oscillation patterns, it proved appropriate to apply an extremely low intensity of FR background illumination (0.4 µmol 730–740 nm quanta/(m^2^s)). The time intervals for assessment of F_0_ and F_m_^ST^ were defined from 0.2 to 0.25 µs and from 1.3 to 1.4 µs, respectively. The result is presented in Fig. 10a and for comparison in Fig. 10b the original data of Delosme (1971a,b) are shown, as reproduced from Fig. 5 of Delosme and Joliot (2002).

**Fig. 11:**
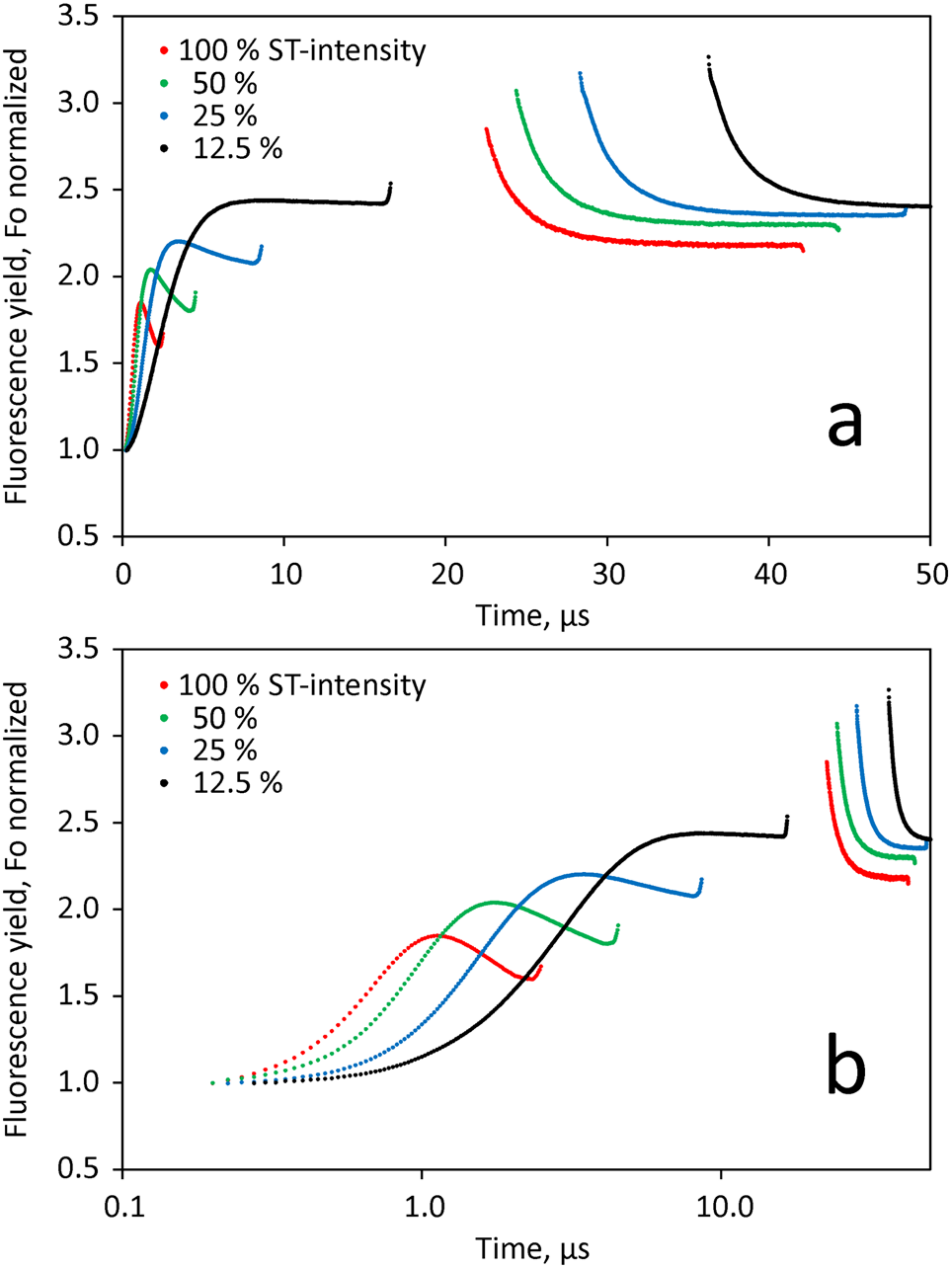
Effect of ST-intensity on the fluorescence yield measured during a saturating ST#1 and 20µs later during a saturating ST#2. 100% intensity resulting in fluence of 1.6 × 10^14^ 440nm quanta cm^−2^ pulse^−1^. As pulse-width was increased in proportion to decreasing intensity, equal fluence with all four pulses. Dark-adapted Chlorella, 22°C. F_0_ normalized and twin ST profile corrected data. In all measurements ST#2 was applied with the same width of 20µs and 12.5% of maximal ST-intensity 20µs after ST#1. Panel a, linear time scale. Panel b, logarithmic time scale

With the new device the oscillation patterns of both F_0_ and F_m_^ST^ obtained via STKS measurement are almost equal to those presented in the pioneering reports of Delosme (1971a, b). In particular, F_m_^ST^ shows maxima in ST#1, 5, and 9, where minima of F_0_ are observed. On closer inspection, however, an important difference between the two data sets is revealed when the amplitudes of the oscillations are compared. For example, the difference between F_m_^ST^ #1 and #3 amounts to 0.32 F_0_ units, whereas in Delosme (1971a, b) it is 0.75 F_0_ units. Hence, if we trust our own data, the oscillation amplitude reported in Delosme (1971a,b) appears to be overestimated by a factor of 2.3. The apparent discrepancy can be resolved by taking a look at the original STK recordings, analysis of which resulted in the F_m_^ST^ (Q_A_^−^ state) and F_0_ (Q_A_ state) data that are plotted in Fig. 10a. The original recordings of STK#1 to STK#6 are presented under Supplementary Materials S4. They display strongly quenched F_v_ and severe distortions compared to ‘normal’ STK recordings, measured at lower ST-intensity, as e.g. shown in the top panel of Fig. 5 (Materials and Methods), where a 20 times weaker ST with a 20 times larger width was applied. While the data of Delosme (1971a,b) are bound to be affected by the same quenching and distortions, these were not apparent because a different measuring technique was applied that did *not provide* any information on the kinetics of the flash-induced changes of fluorescence yield:

1. René Delosme measured F_m_^ST^ via the integral of the ST-excited fluorescence during a 1 µs time window 0.5 µs after full reduction of Q_A_. As apparent from our data in Fig. 4c and in Supplementary Materials S4, this F_m_^ST^ is strongly suppressed by HIQ. This aspect will be elaborated on in more detail in the experiment of Fig. 11 below.
2. For assessment of F_0_ he applied a weak detecting flash. Consequently, the scales of the two types of flash measurements had to be somehow ‘standardized’. In contrast, in the case of our STK measurements F_0_ and F_m_^ST^ are assessed by *one and the same recording* and, hence, with *identical scaling*.
3. For standardization of the two scales, he determined maximal fluorescence yield in the presence of DCMU and strong continuous background light both during a saturating ST and using weak detecting flashes.
4. Doing so, he explicitly assumed that the yield of maximal fluorescence does *not* depend on flash intensity. In reality, however, as already suggested by Schreiber et al. (2019) and will be confirmed below (see sub-section on ‘Lowering of F_m_^ST^ by HIQ’), quenching of F_m_^ST^ by HIQ is an increasing function of quantum flux density.
5. He then assumed that the ratio of F_m_^ST^ / F_0_ equals the ratio of F_m_ / F_0_ measured with weak detecting flashes, which in view of the strong quenching of F_m_^ST^ leads to severe overestimation of the changes in F_m_^ST^, when these are expressed in F_0_ units.
6. In reality, the fluorescence yield of F_m_^ST^ is *not* equivalent to that of F_m_ measured with weak detecting flashes, since, at high quantum flux densities even in the presence of DCMU the fluorescence yield is suppressed by HIQ (Schreiber et al. 2019). The amplitude of the S-state dependent changes of F_m_^*ST*^ is very relevant in the evaluation of the differences in the F_m_/F_0_ ratio that is obtained using ST- and MT-protocols in the experiments of Figs. 7 and 8.

### ***Lowering of F***_***m***_^***ST***^*** by HIQ***

The lowering of F_v_ and F_m_^ST^ by HIQ was investigated in some more detail by twin STK measurements. In the experiment of Fig. 11, twin STK of dark-adapted *Chlorella* were measured at 4 different relative intensities of ST#1 (100, 50, 25 and 12.5% of maximal intensity). As outlined under Materials and Methods, the absolute quantum flux density at maximal intensity was about 1.3 mol 440 nm quanta/(m^2^s). The four STK recordings were normalized at F_0_ = 1. The *integrated light energy* applied at the various intensities was rendered equal by programming ST#1 widths of 2, 4, 8 and 16 µs, respectively. Hence, in all measurements the same fluence of about 1.6 × 10^14^ 440 nm quanta cm^−2^ pulse^−1^ was applied, which saturates turnover of PSII in a dilute suspension of *Chlorella* (see section on ‘Saturation of ST-induced variable fluorescence yield’) below.

For assessment of HIQ relaxation, ST#2 was given 20 µs after termination of ST#1 (see section on ‘Twin flashes for measuring dark relaxation, assessment of HIQ and determination of F_m_^ST^’ under Materials and Methods). In all measurements the same relative intensity of ST#2 (12.5% of maximal intensity) with 20 µs ST-width was used and the same dark-time of 20 µs was applied between ST#1 and ST#2.

From the data in Fig. 11 it is clear that the STK measured at high ST-intensities is dramatically distorted by HIQ. At the lowest intensity (black curve, 12.5% of maximal intensity), the rate of increase of fluorescence yield due to Q_A_ reduction (during ST#1) approximately matches the rate of yield decrease caused by HIQ development (apparent during ST#2), resulting in a seemingly ‘normal’ rise curve. However, the ST#2 response reveals that during the course of this rise 37% of F_v_ was suppressed by rapidly reversible HIQ (relaxation within 20 µs dark-time). With increasing ST-intensity, HIQ increased, amounting to 66% of F_v_ at maximal intensity (red curve). In parallel with the fluorescence rise becoming more and more suppressed, a pronounced dip phase developed. The latter reflects a delay in HIQ formation with respect to the fluorescence rise caused by Q_A_ reduction.

If HIQ were caused by carotenoid triplet quenching (TQ) only, all four F_m_^ST^ values determined by ST#2 20 µs after ST#1 would be expected to be about equal, as at that point of time TQ should be almost fully relaxed. In reality, with decreasing intensity of ST#1 there is a substantial increase of F_m_^ST^. This increase may be explained by the following rationale: During ST#1 at all intensities RCII is first transformed from state **S**_**1**_** Yz P680 Q**_**A**_ to state **S**_**1**_** Yz**^**+**^** P680 Q**_**A**_^**−**^ in equilibrium with **S**_**1**_** Yz P680**^**+**^** Q**_**A**_^**−**^, with the latter causing DQ. Upon the following reduction of Yz^+^ by the OEC, the non-quenching state **S**_**2**_** Yz P680 Q**_**A**_^**−**^ is formed with t_1/2_ = 70 µs (Dau and Haumann 2007). The same t_1/2_ applies for the relaxation of DQ. Formation of the non-quenching state not only occurs during the 20 µs dark-interval between ST#1 and ST#2, but also *during* ST#1. While the latter is negligible in the case of the 2 µs ST, it grows to an appreciable extent in parallel with ST-width. At the end of ST#1 the fluorescence yield is non-photochemically quenched by a composite of TQ and DQ.

The above considerations are facilitated by the fact that at the given time scale just one charge separation per PSII may be assumed, so that mixing of S-states is minimized. In this way, very detailed information on the reactions at the PSII donor-side can be obtained. On the other hand, the information on the rate of Q_A_ reduction that is obtained due to suppression of photochemical quenching (photochemical rise) is severely distorted by HIQ. For the study of the photochemical rise, ST width of 20–40 µs at 5–10% of maximal ST-intensity appear most suitable, where HIQ formation is moderate and double hits still are unlikely.

### Saturation of ST-induced variable fluorescence yield

The lowering of F^ST^ by HIQ (i.e. by a combination of TQ and DQ) complicates quantitative studies on the relationship between ST-intensity and the ST-induced increase of fluorescence yield (saturation curves of F_v_^ST^). For *quantitative* assessment of F_v_^ST^, both forms of HIQ have to be completely relaxed and the dark-time ∆t between the ST and the measurement of F_v_^ST^ must be chosen such that PSII reaction centers cannot reopen, neither by the forward reaction (Q_B_ reduction) nor via recombination (i.e. re-reduction of P680^+^ by Q_A_^−^). The latter occurs in the 100-200 µs time range (Renger and Wolff 1976; Havemann and Mathis 1976) and is enhanced whenever the lifetime of P680^+^ extends into this range of time. In samples with a fully competent OEC, the re-reduction kinetics of P680^+^ are fast and multi-phasic. They are dominated by electron transfer from Y_Z_ to P680^+^ in the nanosecond time domain (Brettel et al. 1984; Eckert and Renger 1988) and in the microsecond time domain by pH-dependent relaxation reactions that are involved in the establishment of the equilibrium between the states P680^+^ Y_Z_ and P680 Y_Z_(ox), coupled with H^+^ movement in the environment of Y_Z_(ox) (Schilstra et al. 1998; Christen and Renger 1999; Christen et al. 1999). In addition to this multi-phasic forward reaction, P680^+^ can also be reduced by Q_A_^−^ via the above mentioned recombination reaction.

**Fig. 12:**
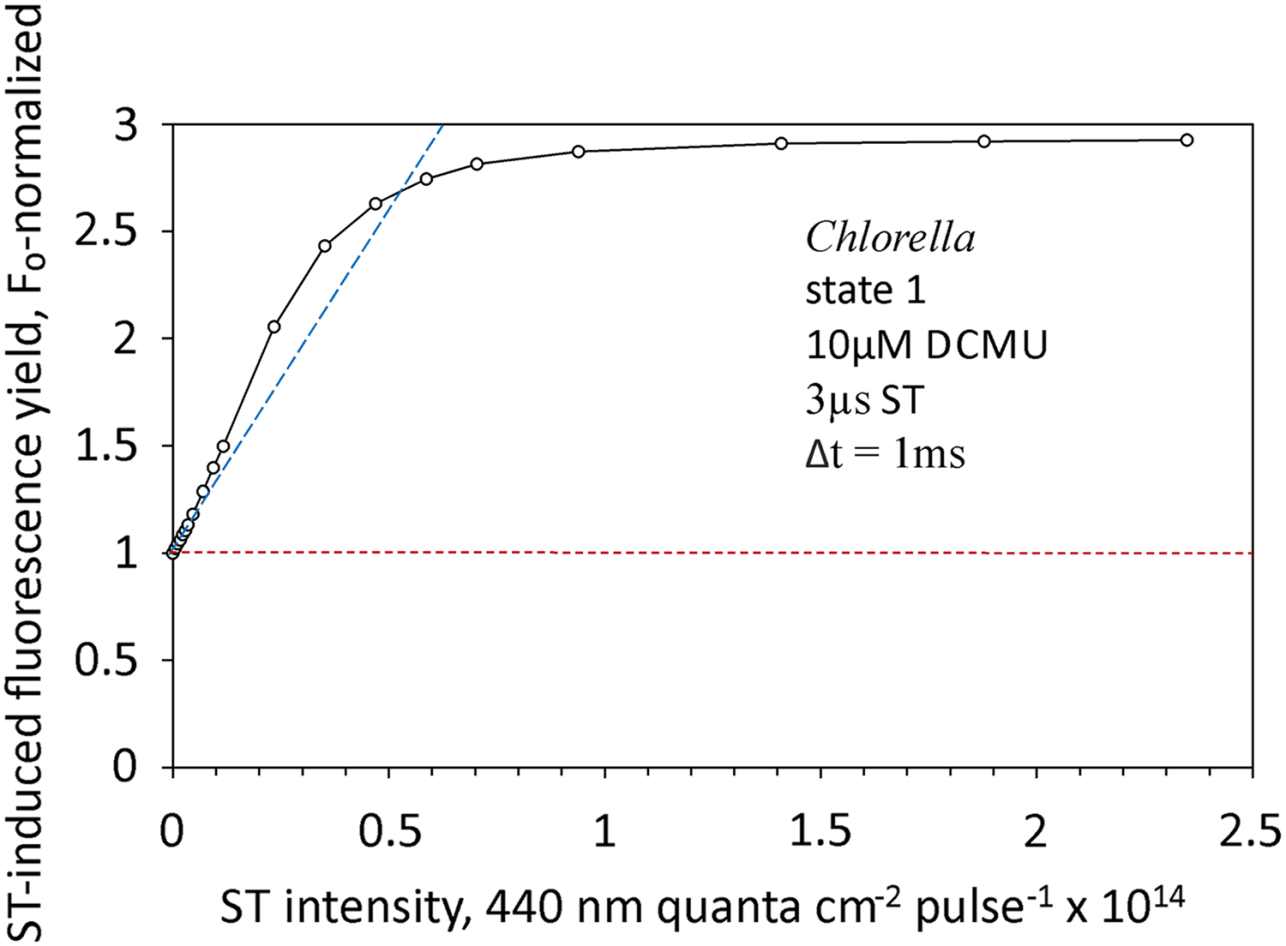
Relationship between the ST-induced increase of relative fluorescence yield and the intensity of a 3µs 440nm ST (pump flash), as measured with a dilute suspension of Chlorella in the presence of 10µM DCMU. State 1 induced by 0.2 µmol 730–740 nm quanta/(m^2^s) background light. ST-induced increase of fluorescence yield measured after 1ms dark-time via ST-triggered Fast Kinetics PAM recordings. For further details, see text and Supplementary Materials S5)

Mauzerall (1972) introduced the pump & probe approach for investigating flash-induced changes in relative fluorescence yield. France et al. (1992) applied this approach for an extensive study of the relationship between ST-intensity and F_v_^ST^, with particular attention to the sigmoidicity in the low fluence range of the light saturation curve. Sigmoidicity was found to be lost, when *at constant integrated flash energy* the flash width was decreased from 50 to 2 µs. Based on a model derived by Valkunas et al. (1991), France et al. (1992) came to the conclusion that the sigmoidicity is caused by sequential two-hit photochemistry in PSII. Hemelrijk and van Gorkom (1992) as well as Lavergne and Rappaport (1998) questioned this interpretation, as they could not find any differences in the saturation curves measured with saturating nanosecond laser or microsecond xenon flashes. As far as we know, thereafter no serious attempts have been made to reproduce the data of France et al. (1992) and to resolve the controversy. While in principle our new device offers itself for such an attempt, this would go beyond the scope of the present communication. Here we just want to demonstrate that the new measuring system is well suited for pump & probe measurements similar to those of France et al. (1992). In Fig. 12 an ST-intensity saturation curve of variable fluorescence yield in the presence of 10 µM DCMU is shown which is analogous to that displayed in Fig. 7A of France et al. (1992).

For the experiment of Fig. 12 a dedicated routine (Script) was programmed, with which the ST intensity settings are automatically varied and a Fast Kinetics PAM recording is triggered at each intensity setting. This means that here pulse-modulated ML is used to determine the increase of fluorescence yield induced by the actinic ST, thus replacing the probe flashes in France et al. (1992). Hence, instead of a single fluorescence value for a fixed dark-time between pump and probe flash, a continuum of data points after triggering of the actinic ST is obtained (for details, see Supplementary Materials S5).

In the given example, the fluorescence signals after 1 ms dark-time were assessed, i.e. after relaxation of HIQ and before reoxidation of Q_A_ via the backreaction in presence of DCMU (Bennoun 1970, Schreiber and Krieger 1996). The resulting ‘saturation curve’ displays pronounced sigmoidicity in the low fluence range, in spite of the fact that 3 µs flashes were used. While this agrees with the findings of Hemelrijk and van Gorkom (1992) as well as of Lavergne and Rappaport (1998), it calls for a reevaluation of the data of France et al. (1992) and their interpretation. Actually, based on model calculations Valkunas et al. (1997) already concluded more than 25 years ago that singlet–triplet annihilation is “a more natural explanation for the observations of France et al. (1992) than the two-hit model of Valkunas et al. (1991)”. This explanation is in line with our findings on the lowering of fluorescence yield by HIQ (see preceding section). It makes sense that an exciton that “escapes” from a closed PSII unit can be either trapped by a neighboring open PSII unit or annihilated via the TQ-mechanism. The probability for the annihilation may be expected to increase with ST-intensity (i.e. quantum flux density), proportionally to which the density of singlet and triplet (via intersystem crossing) excited states of Chl increases.

### S-state advancement induced by weak FR in Chlorella

**Fig. 13:**
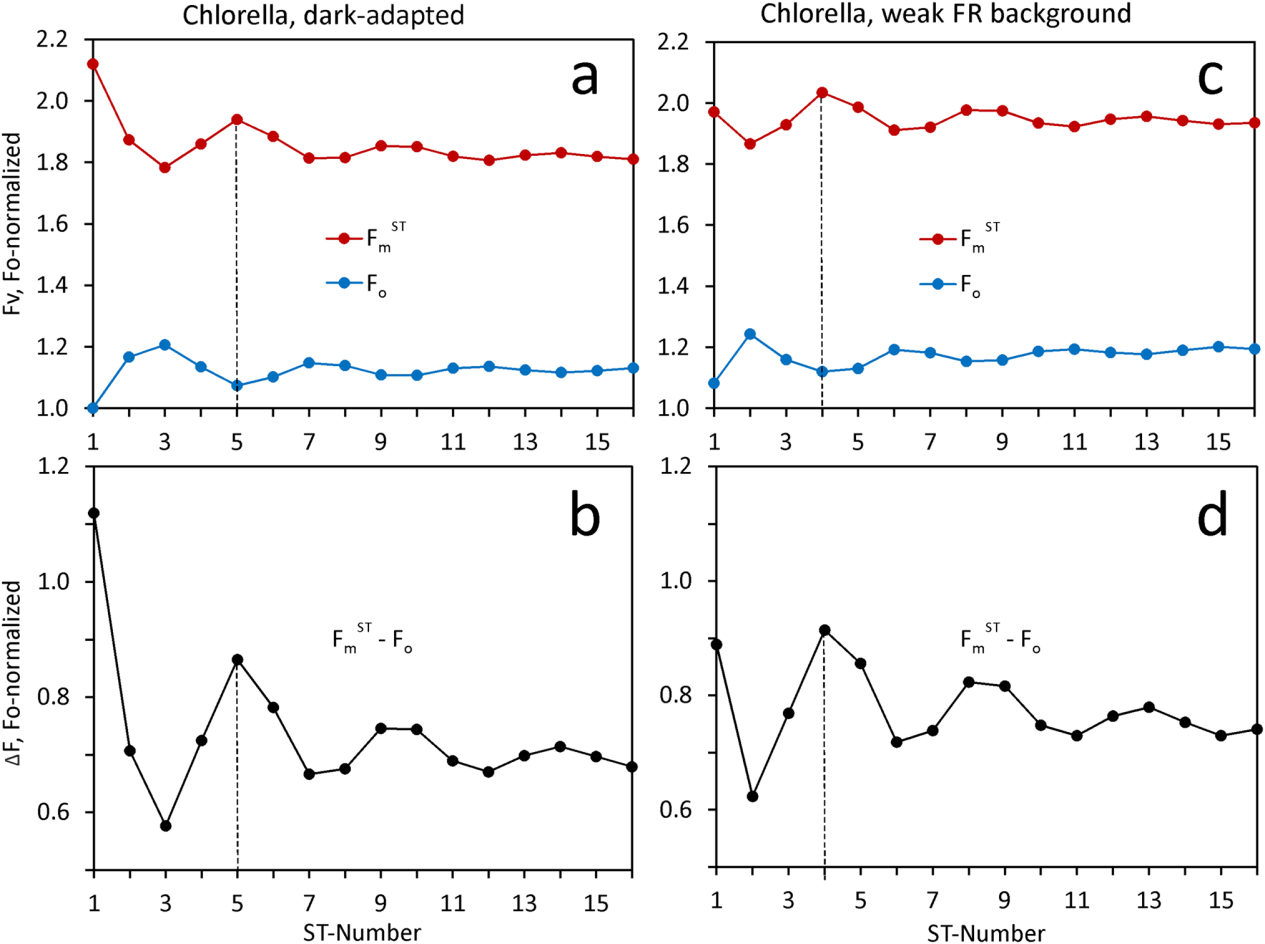
Comparison of STKS measured after extended dark-adaptation in the absence (panels a and b) and presence (panels c and d) of weak FR background light at 2 µmol 730–740 nm quanta/(m^2^s). Predawn Chlorella. Ten consecutive 2µs ST at maximal intensity, repetition time 200 ms. ST profile corrected. Interval analysis: F_0_ at 0.25 -0.3 µs; Fm^ST^ at 1.0–1.1 µs

As already apparent by comparison of the data presented in Figs. 7 and 8, weak FR background illumination has a surprisingly large effect on both the in vivo polyphasic kinetics of the MT-induced fluorescence rise and the STK. This is particularly true for experiments with predawn *Chlorella*, where due to a low level of stroma reductants, the dark rate of PQ reduction is low, so that a rather low quantum flux density of PSI light suffices to oxidize the PQ pool. In Fig. 8 it was shown that weak FR not only causes lowering of the I_1_-level, but of F_m_^ST^ as well. In Fig. 13 evidence is presented suggesting that the lowering of F_m_^ST^ goes along with an apparent advancement of the S-state distribution by one step. As mentioned in connection with the data in Figs. 7 and 8, also the S-state advancement is not only observed with weak FR, but with weak 540 nm background light as well (see Supplementary Materials S6).

Comparison of panel a (dark-adapted) and b (weak FR background) reveals a rather specific effect of the FR on F_0_ and F_m_^ST^ measured in STK#1. Phenomenologically, it is as if in the dark-adapted sample the weak FR can bring about a similar change as ST#1. While in the absence of FR background light, maximal F_m_^ST^ and minimal F_0_ are observed in STK#5, with FR preillumination maximum and minimum, respectively, already are observed in STK#4. This effect is even more clearly revealed in the difference plots of F_m_^ST^—F_0_ presented in panels b and d.

Based on the Kok model of water-splitting, i.e. the linear four-step accumulation of positive charges at the donor side of PSII (Kok et al. 1970), in principal the FR effect may be explained in two different ways:

1. If it is assumed that *Chlorella* after thorough dark-adaptation displays a significant population of S_0_, this would be shifted to S_1_ by weak FR.
2. If *Chlorella* were in state S_1_ after thorough dark-adaptation, this would be shifted to S_2_.

At present, no means is available for unequivocal assessment of the S-state distribution by fluorescence measurements alone. It appears plausible, however, to assume that minimal F_m_^ST^ is observed when the system is in S_3_ before the ST, as before stabilization of the transition to S_0_, the redox equilibria at the PSII donor side are maximally shifted towards the non-photochemical quencher P680^+^. If this assumption is correct, after dark-adaptation the system would have to be in S_1,_ which with weak FR background illumination is shifted towards S_2_.

We note that the F_v_ (or ∆F) induced by ST#1 in thoroughly dark-adapted samples is much larger than in following ST in the sequence. In view of the fact that the same effect can be induced not only by weak FR, but by weak 540 nm as well, one might suspect a role of so-called “inactive PSII”, in which the usual Q_A_-Q_B_ electron transfer is disabled, because the Q_B_ binding site is vacant or temporarily blocked by the presence of Q_B_H_2_ (Lavergne and Leci 1993; de Wijn and van Gorkom 2001; Schansker and Strasser 2005). Under Supplementary Materials S6 preliminary data are presented showing that weak 540 nm, which causes an S-state advancement, causes a rapid fluorescence increase, likely to reflect closure of “inactive PSII”. The weak FR, however, which causes an equal S-state advancement, induces hardly any rapid fluorescence rise, i.e. it does not contain sufficient quanta absorbed by PSII to close “inactive PSII”. This conclusion was confirmed using the following experimental approach:

Measurements of PAM Fast Kinetics allow to determine the rate of wavelength-dependent absorption of photosynthetically active radiation in PSII, PAR_II_ (in quanta/(PSII * s), in dilute suspensions (Schreiber et al. 2012; Klughammer and Schreiber 2015). When rapid Q_A_ reoxidation is blocked by DCMU, such measurements can also be carried out using the maximal available FR-intensity, which is 300 times higher than the intensity used for FR background illumination in the experiments of Figs. 8 and 13. In the presence of DCMU, using maximal FR intensity PSII is closed within about 1 s.Using 540 nm light, the intensity can be determined that is required to obtain the same initial rate of the fluorescence rise as with maximal FR intensity, so that the 540 nm and FR intensities are known, at which equal rates of PSII excitation are obtained. Then these intensities can be compared with the intensities that induce a similar S-state advancement. The results show that 1/300 of maximal FR intensity gives a similar S-state advancement as 1/40 of the 540 nm light that is equivalent to maximal FR intensity in terms of PSII activity (not shown in the figures). Hence, for S-state advancement by weak FR its PSI action appears to be decisive.

### S-state advancement induced by weak FR in a dandelion leaf

The Multi-Color-PAM originally was developed for advanced PAM fluorescence measurements with dilute suspensions of algae and cyanobacteria (Schreiber et al. 2012). However, as recently demonstrated in a study of variable PSI fluorescence, F_**v**_(I), it also can be applied for advanced studies of leaf fluorescence (Schreiber 2023). In this case, using the original Multi-Color-PAM, the leaf may replace the cuvette in the center of the Optical Unit, being positioned at 45° angle with respect to the Multi-Color Emitter and Detector units. In addition, as depicted above in Fig. 1b, the extended version of this instrument now provides a dedicated Emitter-Detector unit for highly sensitive measurements of chlorophyll fluorescence *from the surface* of oblique photosynthetic organisms, like leaves, lichens, macroalgae and corals. While a comprehensive description of such measurements would go beyond the scope of the present report, here just a typical example of application with a dandelion leaf is presented. Making use of a leaf cuvette with temperature control and gas through flow, that originally was developed for gas exchange measurements (Walz, 3010-S) the leaf can be maintained for longer periods of time in a physiologically healthy state.

**Fig. 14:**
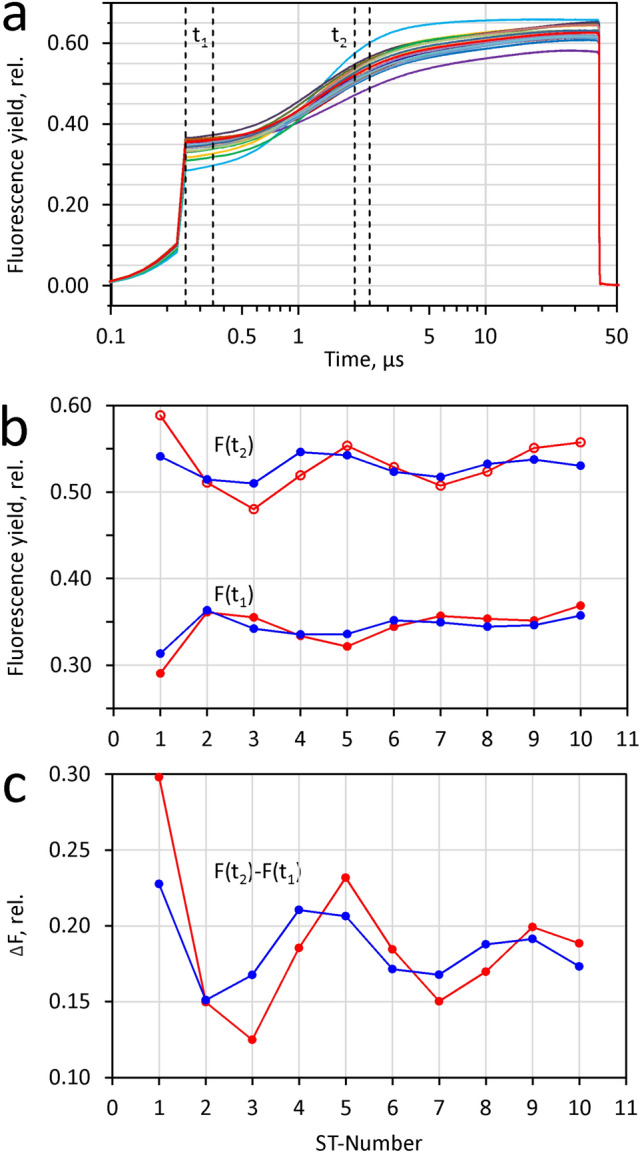
Effect of weak FR background light on STK sequence measured from the adaxial side of an intact dandelion leaf using the ST-Emitter-Detector unit. Panel a, superimposed original STK responses, with definition of the time intervals t1 and t2 for determination of F0, F(t1), and FST, F(t2), respectively. Panel b, plots of F0, F(t1), and FST, F(t2) as a function of ST-Number; red symbols, dark-adapted with single 40µs saturating ST applied 3 min before STKS measurement; blue symbols, dark-adapted sample exposed for 10min to weak FR background light at 2 µmol 730–740 nm quanta/(m2s). Panel c, difference between FST and F0 derived from the data in panel b

As shown in the experiment of Fig. 14, the effect of weak FR on the STK measured from the adaxial surface of a dandelion leaf is similar to that described in Fig. 13 for a suspension of *Chlorella*. The responses elicited by a sequence of ten consecutive saturating 40 µs ST with 100 ms dark-time between consecutive flashes in the presence and absence of weak FR background light are depicted. In the latter case, 3 min before STKS recording a single saturating 40 µs ST was applied in order to make sure that any PSII that after dark-adaptation was in the S_0_-state, is transformed into the S_1_-state. In panel a the original STK are displayed and the two time intervals are indicated during which the F_0_ and F^ST^ values are assessed. These are plotted in panel b against the ST-Number. In panel c a plot of the difference between F^ST^ and F_0_ is presented.

Similarly as shown in Fig. 13 for *Chlorella*, there is an advancement of the initial S-state distribution by weak FR background illumination also in the dandelion leaf. Notably, no such advancement was induced by the saturating 40 µs pre-flash applied 3 min before STKS recording in the absence of FR, suggesting that an intact dark-adapted dandelion leaf displays close to 100% S_1_. We note, however, that in separate experiments with other leaves (e.g. coffee) substantial dark populations of S_0_ were revealed by STKS measurements (data not shown). Furthermore, also in the case of the dandelion leaf a very similar phenomenology with S-state advancement was not only observed using weak FR but weak 540 nm background light as well (not shown in the figures).

## Concluding discussion

Measuring chlorophyll fluorescence has been a pioneering tool in photosynthesis research, since more than 90 years ago Hans Kautsky discovered the phenomenon of light-induced changes of chlorophyll fluorescence (Kautsky and Hirsch 1931). Before discussing the results of the present report, some milestones in the progress of this tool shall be outlined which have stimulated the development of our new device. Since Kautsky’s discovery, fluorescence measurements have become more and more sophisticated, taking advantage of the continual technical progress in the sensitivity and time-resolution of detecting devices as well as the intensity of the excitation sources, including various types of flash lamps. For his pioneering work on the polyphasic rise kinetics, René Delosme made use of a continuous xenon-arc light source, obtaining a 5 µs light-on profile using an extraordinary ‘bullet-shutter’ (Delosme 1967). It may be mentioned that a very similar ultra-fast mechanical shutter was developed by Hoffmann (1967) in the laboratory of Ulrich Franck (Aachen, Germany), which the senior author of these lines applied during his Ph.D. studies. Only 4 years later René Delosme made the next pioneering discovery, when he succeeded to measure the fluorescence yield *during* a 2 µs xenon-discharge flash (Delosme 1971a,b) and then observed the S-state dependent period-4 oscillation of maximal fluorescence yield. Thereafter, Xe-flash devices together with powerful and ultra-fast lasers for several decades have been the main tool for elucidation of the complex electron transfer reactions at the PSII donor- and acceptor-sides.

The introduction of pump & probe flash measurements of relative fluorescence yield by David Mauzerall has led to considerable progress in photosynthesis research (Mauzerall 1972, 1976; Lavergne and Rappaport 1998; Schilstra et al. 1998; Christen et al. 1999; Steffen et al. 2001; de Wijn and van Gorkom 2002).

Pioneering studies on high intensity fluorescence quenching *during* strong pulses of light were carried out by Zankel (1973), Duysens and coworkers (den Haan 1976; Sonneveld et al. 1979) and by Breton et al. (1979).

After introduction of PAM fluorimetry (Schreiber 1986; Schreiber et al. 1986), Xe-flashes have been used for defined ST-preillumination, whereas fluorescence yield has been measured with pulse-modulated ML from light-emitting-diode (LED) sources. In this way, Schreiber and Neubauer (1987) were able to show that the I_1_-level of the polyphasic fluorescence rise displays the same period-4 oscillation pattern that was described by Delosme (1971a, b). Trtilek et al. (1997), Nedbal et al. (1999) and Koblizek et al. (2001) demonstrated that it is not only possible to generate saturating 20-100 µs ST with an LED-array, but also to *measure the kinetics of the fluorescence increase during* such an ST, which carries valuable information on the optical cross-section of PSII. Kolber et al. (1998) introduced the ‘fast repetition rate’ fluorimetry (FRRF), based on LED-flashes as well, where a large number of ‘flashlets’ (0.2–0.8 µs width, with variable repetition times) are employed to provide the cumulative excitation energy equivalent to an ST for full closure of PS II reaction centers and for measuring the fluorescence rise kinetics *during* this ‘functional ST’. A modified FRRF-version based on 1 µs flashlets generated by a laser-diode was developed by Ananyev and Dismukes (2005) for highly sophisticated simultaneous measurements of period-4 oscillations of fluorescence yield and ST-induced oxygen evolution.

The new instrument, the technical features and performance of which are described in the present report, is unique in enabling comparative and even simultaneous PAM- and flash-kinetics measurements. This combined measuring system provides for unprecedented versatility and flexibility in the assessment of fluorescence yield. It offers a large variety of differently colored ML, AL, MT and ST for measurements in dilute suspensions as well as in whole leaves. Both MT- and ST-protocols can be used for determination of maximal fluorescence yield and calculation of fluorescence-based photosynthetic parameters. The data presented above focus on five major aspects:

1. performance of measurements of flash-kinetics in terms of signal quality, time resolution, flexibility in repetition rates and reproducibility
2. significance of the difference between the maximal fluorescence yields observed upon application of MT and ST
3. assessment of quenching by carotenoid triplets (TQ) that lowers fluorescence yield during an ST and rapidly relaxes after an ST
4. period-4 oscillations in fluorescence yield that are induced in a sequence of saturating ST
5. the surprisingly large effect of weak 730–740 nm light on the flash-kinetics and the period-4 oscillation pattern

Based on the presented data the following conclusions may be drawn:

Ad 1) The signal/noise ratio is exceptionally high, so that generally *no averaging* is required. This aspect is particularly important for in vivo application, where the reproduction of a particular physiological state often is problematic and time-consuming. Even at maximal ST intensity, when Q_A_ is almost completely reduced within about 1 µs and the fluorescence yield is strongly lowered by TQ, the initial fluorescence yield, F_0_, can be reliably determined in the 0.2–0.3 µs range with the help of a dedicated ‘rising-edge correction’ routine (see Figs. 3 and 11). Furthermore, the amplitude and on–off profile of the rectangular-shaped ST is exceptionally constant, displaying less than 10^–3^ variation.

Ad 2) The difference between the maximal fluorescence yields determined by MT and ST pulses depends on the physiological state of the sample. It is minimal after thorough dark adaptation when particular care is taken to prevent any form of ambient background light. A substantial part of this difference, corresponding to the I_2_-P transient of the polyphasic rise, recently was shown to reflect variable PSI fluorescence, F_v_(I) (Schreiber and Klughammer 2021; Schreiber 2023). The remaining difference of 0.15 F_0_ units (Fig. 7) is in the same order of magnitude as the variation of F_m_^ST^ in an ST sequence (Figs.10a and 13a), caused by the S-state dependent period-4 oscillation of DQ. Much larger differences between F_m_^MT^ and F_m_^ST^ in the order of 50% of F_v_ have been reported in the past (Samson and Bruce 1996, reviewed in Samson et al. 1999 (where a variety of different explanations are discussed) and in Garab et al. (2023).

In this context, it has to be considered that it is much more easy to determine F_m_^MT^ than F_m_^ST^. In the case of F_m_^MT^ charge separation is readily blocked by reducing all PSII acceptors, including the PQ-pool, and sufficient time is provided for the relaxation of DQ. On the other hand, in the case of F_m_^ST^, the ST-induced 100% Q_A_^−^ is short lived, so that F_m_ has to be measured as close as possible to the ST, using a probe flash or high frequency modulated ML. In practice, the probe flash should be applied about 20 µs after the saturating ST, when TQ may be assumed to be almost fully relaxed (see e.g. Figure 7). At this point of time, however, fluorescence yield is still affected by DQ, which relaxes in parallel with the reduction of Yz(ox) by the OEC (S-state transition). The extent of DQ depends on the S-state distribution before F_m_^ST^ measurement. As demonstrated in Fig. 7, after thorough dark-adaptation, when a large population of S_0_ + S_1_ may be assumed, F_m_^ST^ is just marginally lower than F_m_^MT^, whereas it is much lower in the presence of weak FR light (Fig. 8), which causes S-state advancement in the initial distribution (Figs. 13 and 14). The observed differences can be readily explained by the largely different transition times of the S-states (see e.g. Dau and Haumann 2007), amounting to 30 µs (S_0_ to S_1_), 70 µs (S_1_ to S_2_), 190 µs (S_2_ to S_3_) and 1300 µs (S_3_ to S_0_), with increasing transition times resulting in longer lifetimes of P680^+^ and correspondingly increasing DQ (Schilstra et al. 1998, Christen et al. 1999; Steffen et al. 2001; Belyaeva et al. 2008).

In the light of these considerations, it appears practically impossible to determine the true maximal fluorescence yield unequivocally using an ST-protocol. When fluorescence yield is measured *during* a saturating ST, it will be partially quenched by TQ and DQ. When it is measured after an ST, TQ can be avoided, but DQ remains. Actually, in the latter case, the relaxation of DQ (increase of fluorescence yield) will overlap with reoxidation of Q_A_^−^ (decrease of fluorescence yield), so that the true maximal fluorescence yield is never reached and has to be estimated by extrapolation.

Ad 3) The TQ that lowers fluorescence yield *during* an ST can be reliably assessed with the help of a second ST applied 20 µs after the first, when ‘twin ST-profile correction’ is applied (Figs. 4 and 11). After being largely relaxed within the 20 µs dark-time, the kinetics of its renewed formation during a second ST can be measured. It is suggested that the annihilation of excited Chl singlet states by carotenoid triplets competes with photochemical trapping and charge-separation. Details on this competition have to be elucidated by further research. The new measuring system provides the means for a thorough investigation of TQ and its role in photosynthesis under in vivo conditions.

Ad 4) In Fig. 10 we have demonstrated that the legendary period-4 oscillations in fluorescence yield measured by René Delosme more than 50 years ago (Delosme 1971a, b) can be almost perfectly reproduced using our new instrument. This is true in spite of profound differences in the applied measuring techniques. We noticed, however, that the scaling in Fig. 1 of Delosme (1971a,b) is distorted by the fact that F_m_^ST^ at the applied ST-intensity is strongly suppressed by TQ. In our measurements, the scaling is reliable, as both F_m_^ST^ and F_0_ are determined from one and the same flash-kinetics. We conclude that the oscillation amplitude in Delosme (1971a,b) is overestimated by about a factor of 2.3, something that may relieve researchers, who during the past 50 years have been trying in vain to reach the same oscillation amplitude as the ‘original’.

While in Delosme (1971a,b) maximal fluorescence yield is measured integratively without resolution of the flash-kinetics, with our new device the *ST-kinetics* (STK) are measured with outstanding signal/noise ratio and time resolution, providing important *additional information*. On closer look at the superimposed STK displayed in Fig. 5a, it is apparent that all curves display a common intersection point. Generally, as depicted in Fig. 5b, an increase of F_0_ correlates with a decrease of F_m_^ST^. These findings point to a common cause of the ST-Number dependent changes in F_0_ and F_m_^ST^, which could be the number of positive charges stored in the OEC. The local field caused by these charges may be envisaged to counteract charge separation, leading to an increase of F_0_, and to shift the equilibrium between Yz(ox) and P680^+^ towards the latter, thus stimulating DQ and decreasingF_m_^ST^. The individual STK in an ST-train, display remarkable differences depending on the ST-Number, a thorough analysis of which would go beyond the scope of this report. These kinetics not only contain information on the rate of primary PSII charge separation but on the rates of the secondary stabilization reactions at the PSII donor and acceptor sides as well. In this context, the widespread assumption that the fluorescence rise in the 20-100 µs time range follows the rate of Q_A_ reduction may be questioned. At the high quantum flux intensities required for closure of PSII within 20-100 µs, formation of DQ cannot be avoided, the relaxation of which will contribute to the fluorescence rise. This finding is not new. Already more than 50 years ago David Mauzerall discovered that after a saturating 25 ns laser flash fluorescence yield is first strongly quenched and then increases in several phases up to about 100 µs (Mauzerall 1972). The multi-phasic P680^+^ reduction kinetics that are responsible for this increase also proceed *during* a 20-100 µs pulse, although less well separated in time (Schilstra et al. 1998, Christen et al. 1999).

Ad 5) The 730–740 nm background light applied in the experiments of Figs. 8, 13 and 14 is so weak that about 40 s continuous illumination are necessary for a single charge separation of PSII. Nevertheless, its effect is dramatic, causing strong lowering of F_m_^ST^ and I_1_, analogously to an S-state advancement by one step. Formally, this means that under otherwise dark-adapted conditions, the initial S-state distribution is shifted from S_0_ + S_1_ to S_1_ or possibly even to S_1_ + S_2_. The most simple explanation relies on the fact that 730–740 nm is much more effective in driving PSI than PSII and that oxidation of the PQ pool favors S-state advancement. A role of Y_D_ (on the D2 protein) may be envisaged, as it is known that S_0_ is slowly oxidized to S_1_ by Y_D_(ox) (Styring and Rutherford 1987; Vass and Styring 1991; Messinger and Renger 1993). Other interpretations may be possible, a thorough investigation of which, however, would go beyond the scope of the present technical report. Some preliminary data are presented under Supplementary Materials S6 on the relationship between FR- and 540 nm induced changes of fluorescence yield in the vicinity of F_0_ (measured via PAM Slow Kinetics) and the period-4 oscillation pattern (measured in defined FR- and 540 nm induced states via STKS recordings). The results suggest that not only the S-state advancement by weak FR but also by weak 540 nm background illumination is driven by PSI via oxidation of the PQ-pool.

In conclusion, the presented data demonstrate that with the extension of the already existing Multi-Color-PAM chlorophyll fluorimeter for measuring flash-kinetics, a powerful new tool has become available for basic and applied photosynthesis research. By combination of PAM and STK measurements novel information can be obtained that has not been accessible before using one of these two approaches alone. In particular, important new insights into the complex regulation mechanisms of overall photosynthesis in vivo may be expected, that involve not only reactions at the level of reaction centers and intersystem electron transport, but at the stroma level as well.

### Supplementary Information

Below is the link to the electronic supplementary material.

Supplementary file1 (DOCX 1929 kb)

## Data Availability

On request, original data can be provided by Ulrich Schreiber.

## Abbreviations

AL: Actinic light
Chl: Chlorophyll
DQ: PSII donor-side dependent quenching of fluorescence yield
F > 700: Fluorescence detected with detector filter set passing wavelengths > 700 nm
F < 710: Fluorescence detected with detector filter set passing wavelengths < 710 nm
F_0_, F_m_: Minimum and maximum fluorescence yield of dark-adapted sample
F(I), F(II): Fluorescence emitted from PS I and PS II pigments systems, respectively
F_v_: Variable fluorescence
F_v_(I), F_v_(II): Variable fluorescence emitted from PS I and PS II pigment systems
F^ST^: Fluorescence signal measured during single turnover flash
F_i_: Initial fluorescence at onset of single turnover flash, in particular of the second flash in a twin ST measurement
F_m_^ST^: Maximal fluorescence yield measured during ST illumination
F_m_^MT^: Maximal fluorescence yield measured during MT illumination
HIQ: High Intensity Quenching of fluorescence yield during ST and MT illumination
ML: Pulse-modulated fluorescence measuring light
MT: Multiple turnover light pulse
OEC: Oxygen evolving complex of photosystem II
O-I_1_-I_2_-P: Characteristic levels of fluorescence yield during the course of the polyphasic fluorescence rise upon transition from the dark to the strongly illuminated state
PAM: Pulse Amplitude Modulation
PAR: Photosynthetically active radiation
PS: Photosystem
RCII: Reaction center of PSII
ST: Single turnover flash
ST#1, ST#2: First and second flash of a twin ST measurement
STK: ST-induced fluorescence rise kinetics corrected for ST-profile
STKS: Sequence of STK measured at defined repetition rate
STK#i: STK measured during ST number i of an STKS
TQ: Triplet quenching constituting a major, rapidly relaxing component of HIQ
