# Supplementary material for: Flash-kinetics as a complementary analytical tool in PAM fluorimetry
Source: Photosynth Res. 2024 May 22;161(3):151–76. doi: 10.1007/s11120-024-01101-w (PMC11324780; doi:10.1007/s11120-024-01101-w)
Supplement: Supplementary file 1 — Supplementary file1 (DOCX 1929 kb) [file 11120_2024_1101_MOESM1_ESM.docx]

Klughammer, Schlosser and Schreiber (2024)

**Supplementary Materials**

**S1 Various types of light pulses provided by the new device**

1. The new ST-lamp in combination with the new Multi-Color Emitter unit provides a large variety of light pulses differing in color, intensity, width, profile and repetition rates.
2. Multi-Color pulse-modulated measuring light (ML) at 440, 480, 540, 590 and 630nm, consisting of 1µs pulses that can be applied at 20 different intensity settings and a total of 14 pulse frequencies ranging from 10 to 10^5^ Hz. At the lowest settings of intensity and frequency this ML does not induce any significant increase of fluorescence yield. When 540nm ML is used, this is true even in the presence of DCMU.
3. Multi-Color actinic light (AL and MT) at 440, 480, 540, 590 and 630nm which may serve for multiple-turnover pulses to induce the polyphasic rise kinetics of fluorescence yield (O-I_1_-I_2_-P) or just the O-I_1_ rise kinetics for assessment of the wavelength dependent absorption cross section of PSII.
4. Multi-color single turnover flashes (ST) at 440nmn, 480nm, 540nm, 590nm, 630nm and white, applied at fixed maximal intensities, the widths of which can be programmed between 2.5 and 50µs. These ST-pulses can be applied with either single color or with all colors together. In the latter case they saturate PSII turnover (Q_A_ reduction) in optically thin suspensions with 40µs ST-width.
5. 440nm ST pulses *originating from the new ST-lamp*, the intensity of which is controlled by the supply voltage of this lamp and can be varied by about a factor of 10^3^ (see Supplementary Materials S2). At maximal intensity the quantum flux density of these ST exceeds that of the 440nm ST provided by the Multi-Color Emitter by a factor of about 45. The width of these pulses can be varied between 0.2µs and 14ms. The below screenshot shows the flash-profiles for 0.2µs to 5µs at max. ST-intensity.


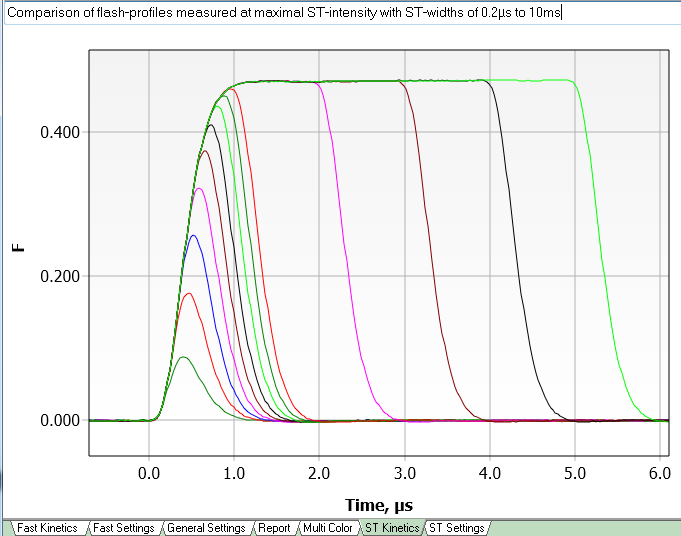


Comparison of flash-profiles measured at maximal ST-intensity (3300mV supply) with ST-widths ranging from 0.2µs to 5µs. ST light scattered from the diffusing white sphere of a spherical micro quantum sensor (US-SQS/WB, Walz) mounted in the center of the cuvette. Single recordings. The relative flash energies (or fluences) are given by the integrated areas below the flash profiles.

**Twin ST and pump-and-probe measurements**

Two consecutive ST (twin ST), characterized by individually defined intensities and widths, can be programmed with defined dark times, t_d_, in between, ranging from 1µs to 14ms. In this way, great flexibility in pump-and-probe measurements is provided, where the pump-flash (pulse 1) normally is stronger than the probe-flash (pulse 2). The latter, however, is not a necessary condition in the case of our new device, as the *ST-kinetics* (STK) of both pulses are measured, so that the dark relaxation from the final state reached in pulse 1 to the initial state in pulse2 can be assessed. Hence, in principle the dark relaxation of fluorescence yield can be measured even when pump and probe pulses have the same intensity.

It is also possible to apply the probe flash repetitively, either with fixed t_d_ or with linearly or exponentially increasing t_d_ (see screenshot below with 11 repetitions and t_d_ increasing linearly from 5 to 55µs).


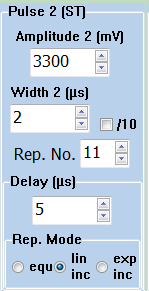


Pulse sequence with 2µs pump-ST and 11x 2µs probe-ST (equal ST-intensities).

Delay between pulse 1 and pulse 2: t_d_ = 5µs

t_d_ linearly increasing from 5µs to 55µs with consecutive probe-pulses


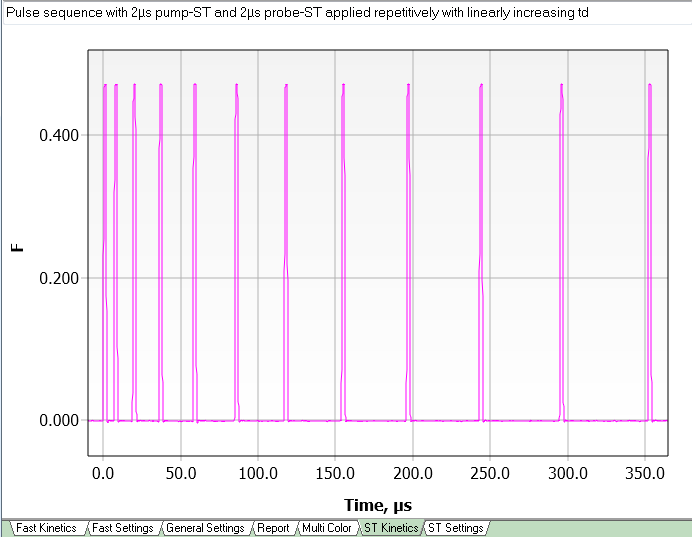


**Triggering of light pulses generated in the Multi-Color Emitter instead of ST-pulses generated by the new ST-lamp**

As mentioned above, at maximal intensity the quantum flux density of the ST generated by the new ST-lamp exceeds that of the 440nm ST provided by the Multi-Color Emitter by a factor of about 45. Therefore, in most applications the former will be preferred to the latter. In some applications, however, the color of light is of primary interest. In this case, the new device offers the possibility to trigger light pulses originating from the Multi-Color Emitter instead of the ST generated by the ST-lamp. This means that the advantages of the new user software developed for control of ST-pulses can be taken advantage of for configurating multi-color light pulses as well. Furthermore, it now has become possible to reliably measure the relative intensities and profiles of the variously colored pulses, making use of the ST-kinetics (STK) detector. Consequently, the relative intensities of multi-color pulses and ST-pulses (generated by the ST-lamp) can be quantitatively compared. For this purpose, use of a spherical micro quantum sensor (US-SQS/WB, Walz) is made which is mounted in the center of the cuvette, with the diffuse white sphere scattering the light from both pulse sources equally towards the STK detector.

The below screenshot shows the responses of six differently colored 40µs ST generated in the Multi-Color Emitter, triggered instead of the ST-originating from the new ST-lamp (Hi-ST).


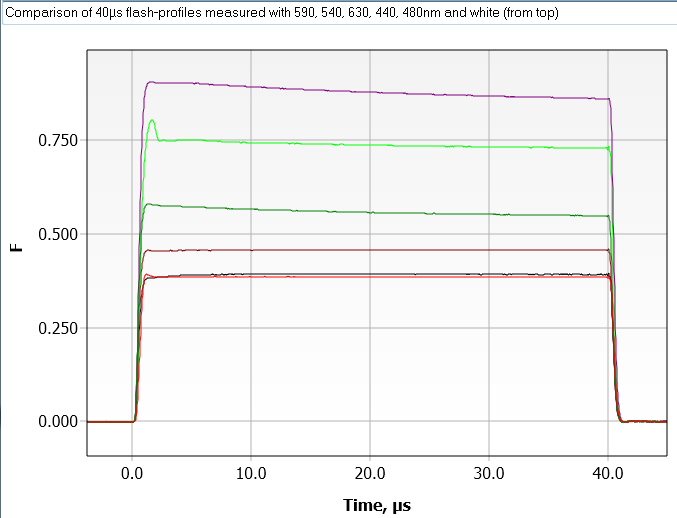


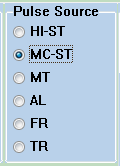

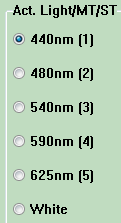


Comparison of 40µs flash- profiles measured with 590nm, 540nm, 630nm, 440nm, 480nm and white (from top).

In the evaluation of the multi-color ST responses displayed in the above screenshot, it should be considered that *relative* intensities are measured, with the signals being convoluted with the spectral sensitivity of the silicon photodiode of the STK-detector. *Absolute* intensities in terms of quanta/(m²s) PAR have to be derived from measurements with longer MT-pulses, the intensities of which can be determined directly with the same spherical micro quantum sensor, as outlined below for 440nm pulses in Supplementary Materials S2.

**S2 Determination of absolute MT- and ST-intensities**

As outlined in the main text under Materials and Methods (sub-section on ‘Flash intensity’), the ST-intensities in the center of the sample in the 10 mm x 10 mm cuvette are determined by comparison with the maximal MT-intensity. While the intensity of a 440 nm MT at maximal setting can be directly measured with a standard spherical micro quantum sensor (US-SQS/WB, Walz), the intensity of a 440 nm ST has to be measured indirectly by comparing the signals generated by the ST and MT light that is scattered at right angle from the diffuse white sphere towards the photodiode in the STK detector (for optical geometry, see main text, Fig. 1a). The user software supports a routine for measuring a list of quantum flux densities of PAR at a given color for 20 intensity settings defined by the user (see screenshot of MT PAR list below).The instrument prototype, with which the measurements of the present study were carried out, reaches 6482 µmol 440 nm quanta/(m²s) at maximal MT-intensity setting 20. As will be shown below, an approximately 200-fold maximal flux density of 440nm quanta is reached with ST-flashes provided by the new ST-lamp.


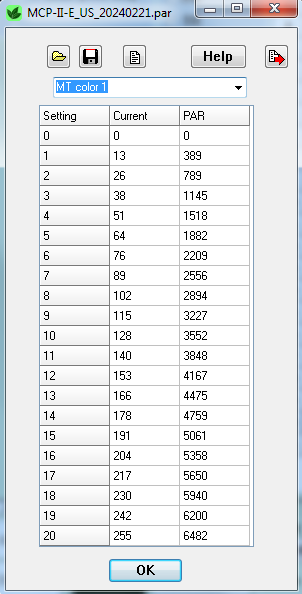


*MT PAR list for the 440 nm MT-intensity settings*

The intensity of the *ST-flashes* is controlled by the supply voltage of the ST-lamp, which can be varied with mV accuracy between 1000 and 3300 mV, resulting in a quasi-continuum of available relative flash intensities ranging from 0.08 % (at 1000 mV) to 100 % (at 3300 mV). 5 % of maximal ST-intensity is obtained at 1267 mV. A list with 21 values of custom supply voltages (ST-list) can be defined. An automated routine is available for first measuring the resulting *relative* ST-intensities (i.e. the signal amplitudes), from which then the *absolute* intensities are calculated in units of µmol (or µE) of 440nm quanta/(m²s). The below screenshot (left) shows the custom ST-list installed for the present study, corresponding to relative intensities 5, 10, 15, 20, 25, 30, 35, 40, 45, 50, 55, 60, 65, 70, 75, 80, 85, 90, 95 and 100 %. In the right screenshot the ST-flash profiles at relative intensities 5, 10, 15, 20, 25, 30, 235, 40, 50, 60, 70, 80, 90 and 100 % are displayed, as measured with the STK detector.


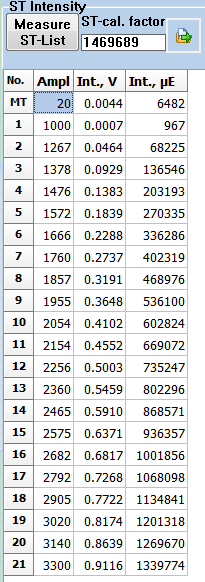

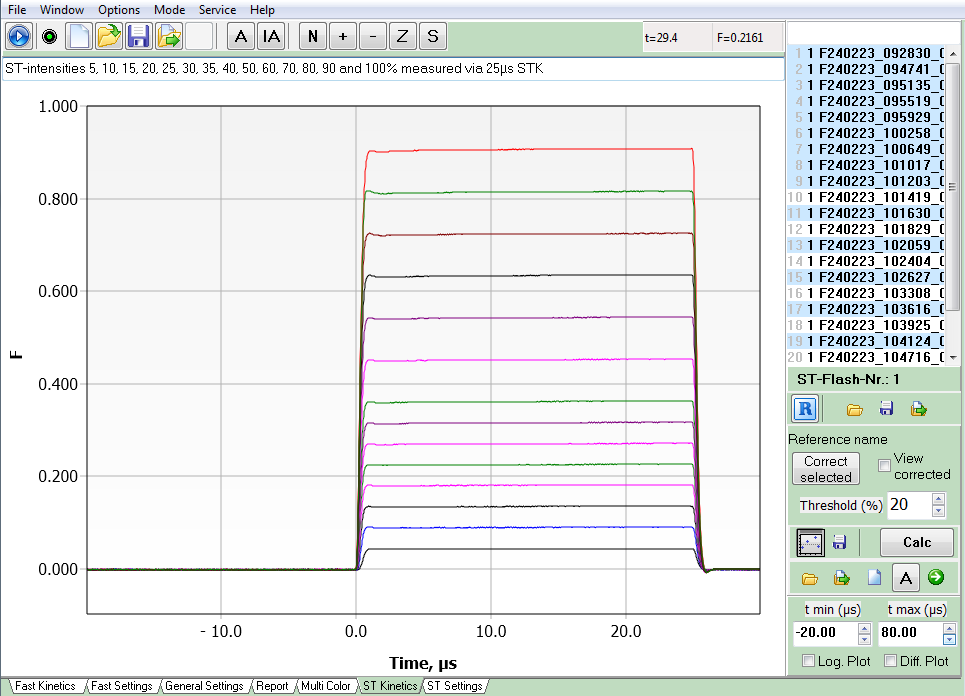


*Custom ST-intensity list* *ST-signals obtained with 14 different supply voltages*

For calculation of *absolute* from *relative* ST-intensities the ratio between the quantum flux density of 440nm MT20 measured with the spherical micro quantum sensor (6482 µmol quanta m^-2^ s^-1^) and the STK-detector signal (0.0044 Volt) caused by the 90° scattered 440nm MT20 is decisive (ST-cal. factor). Maximal ST-intensity (100% with 3300mV supply voltage) amounts to 1.34 mol quanta m^-2^ s^-1^ (or 1.34 E m^-2^ s^-1^, equivalent to 6.02 x 10^23^ x 1.34 quanta m^-2^ s^-1^ or 8.1 x 10^13^ quanta m^-2^ s^-1^. Hence, e.g. maximal fluence of a 3µs ST amounts to about 2.4 x 10^14^ quanta per cm² and pulse.

The custom ST-intensity list can be exported to Excel. The resulting plots of relative and absolute ST-intensities versus ST-supply voltage are presented below.


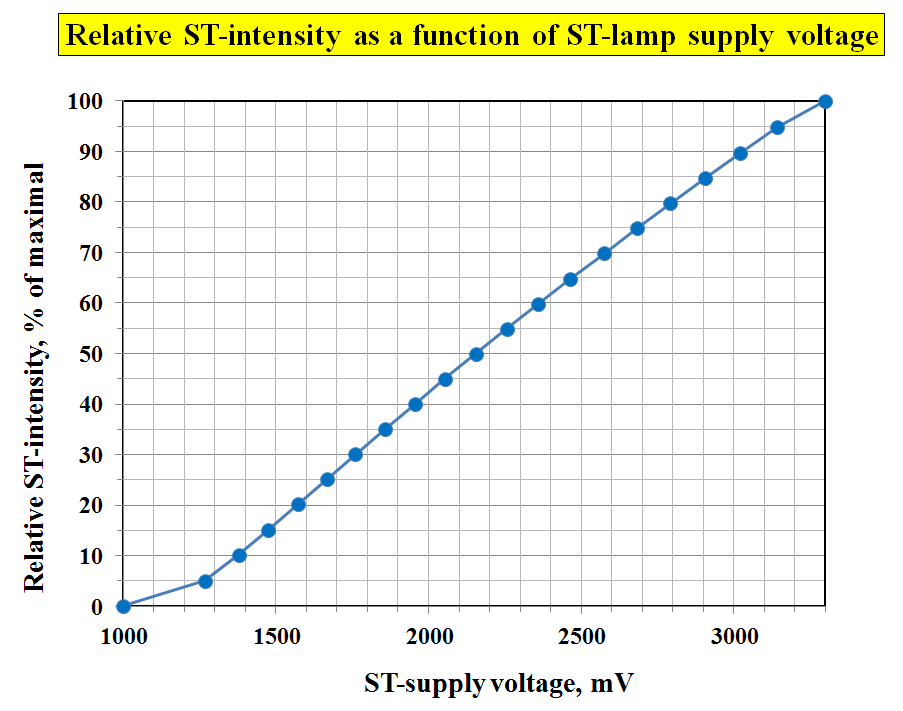


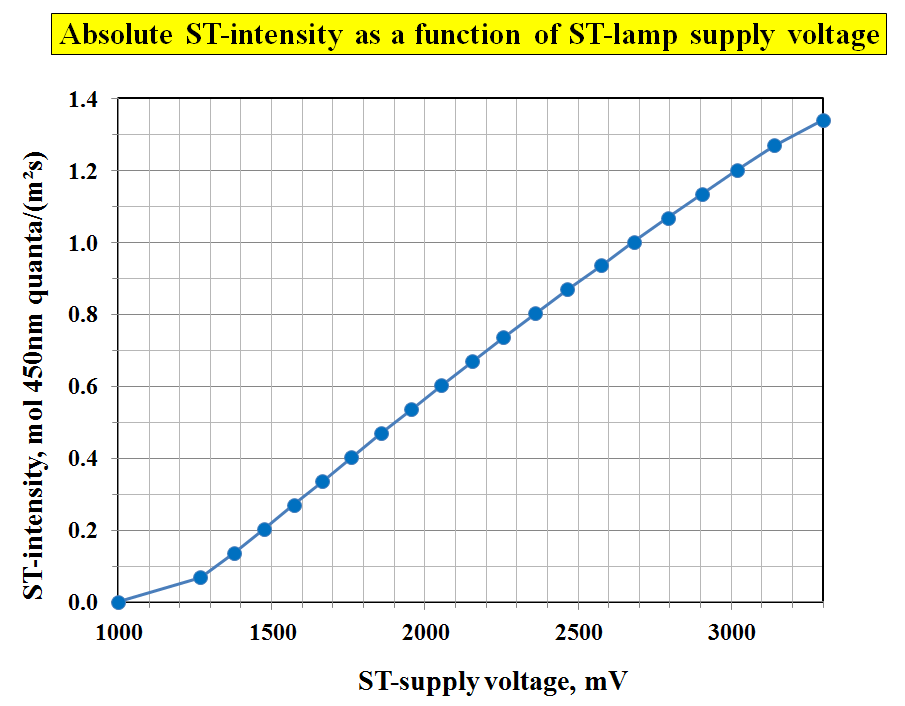


**S3 Triggering of ST-kinetics (STK) simultaneously with PAM ML-pulses and Fast Kinetics recordings**

An important feature of the new measuring system is that it enables to carry out *comparative measurements* of pulse-modulated fluorescence yield (PAM) and ST-kinetics (STK) *on the same sample* in a given physiological state. Hence, for the first time it has become possible to *quantitatively* compare and evaluate changes of light induced fluorescence yield recorded by these two measuring techniques. For this purpose, it is important that an STK can be triggered at a defined point in time of a PAM recording, which characterizes a defined state of the investigated sample. Furthermore, it is essential that after normalization of the relative fluorescence yields measured by PAM and STK, any change in fluorescence yield is “seen” equally by the two approaches. The experiment of Fig. 6 (main text) shows that this indeed is the case. Here some details on the relationship between the intensities and time courses of PAM ML-pulses and the ST-pulse applied in this experiment are given.

For the sake of demonstrating the equivalence of PAM and STK responses, the intensity of a 150µs ST was adjusted such that it closely matched the intensity of individual 1µs PAM ML-pulses, as measured with the STK-detector via the light scattered from the white sphere of a spherical micro quantum sensor (US-SQS/WB, Walz) mounted in the center of the cuvette (see also Supplementary Materials S1 and S2). The 150µs ST generated by the ST-lamp triggered the Fast Kinetics PAM recording at time=0. The screenshot below shows the Fast Kinetics Trigger file used in this experiment.


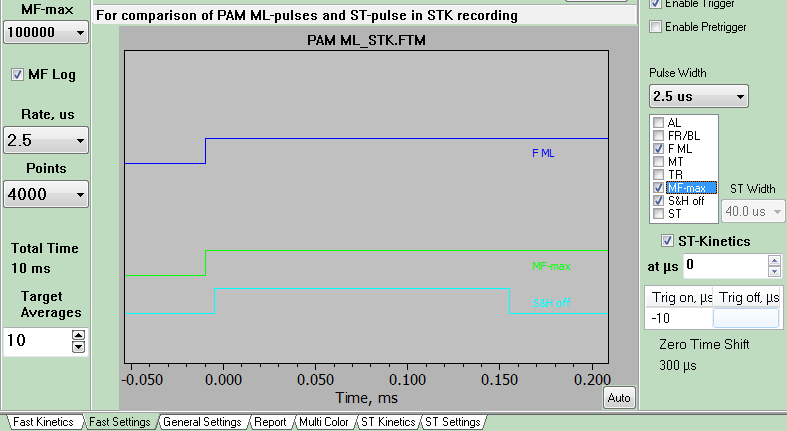


For the given purpose (i.e. the comparison of PAM ML-pulse and ST-pulse intensities) not the PAM recording, but the ST-kinetics recording is of interest, which is shown in the following screenshot. Close to equal pulse amplitudes, reflecting equivalence of quantum flux densities, were obtained at PAM ML intensity setting 11 and an ST-lamp supply voltage of 1083mV.


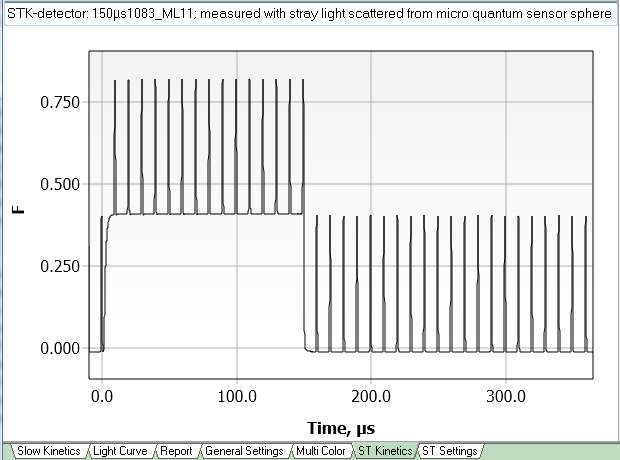


The corresponding original recording of the ST-kinetics is displayed in the below screenshot.


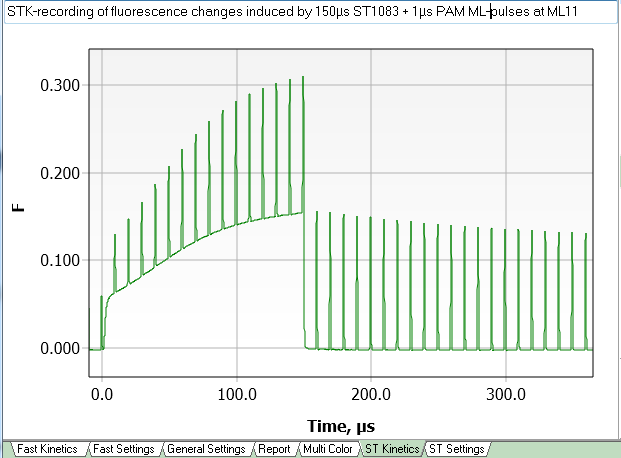


Based on this original recording the Exel-plots presented in Fig. 6 (main text) were derived, which confirm the quantitative equivalence of the changes of fluorescence yield assessed by STK and PAM measurements.

It may be noted that the actual PAM signal is based on the amplitudes of the ML-pulse induced responses (“spikes”) as measured by the PAM pulse detector and a dedicated pulse amplifier. The original pulse responses are transformed into a final continuous PAM signal.

**S4 Original recordings of STK#1 to STK#6 of the experiment in Fig. 10a**

Dilute suspension of *Chlorella vulgaris* at 10°C. The vertical lines define the time ranges within which the data points were averaged for determination of fluorescence yield in the Q_A_ and Q_A_^-^ states.


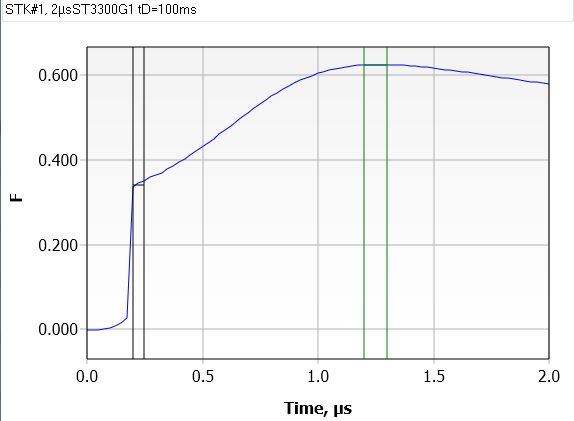
 **STK#1**


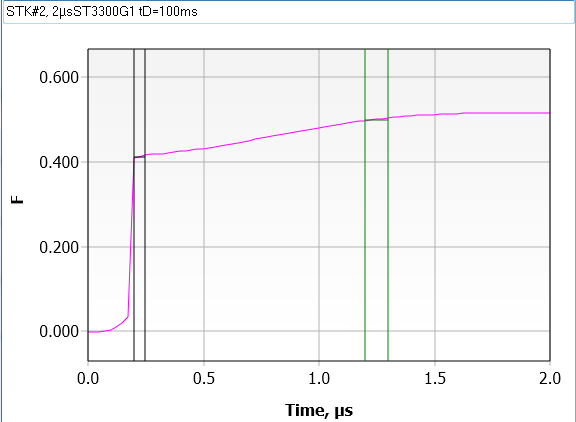
 **STK#2**


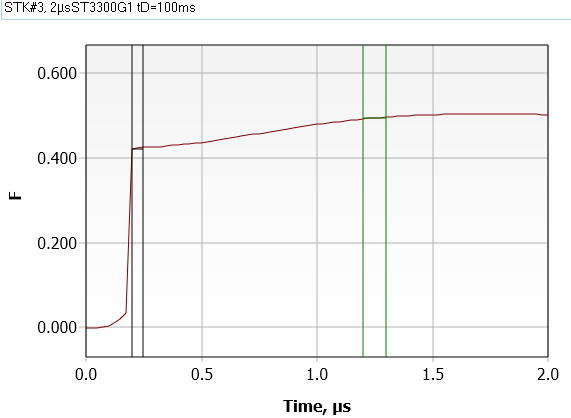
 **STK#3**


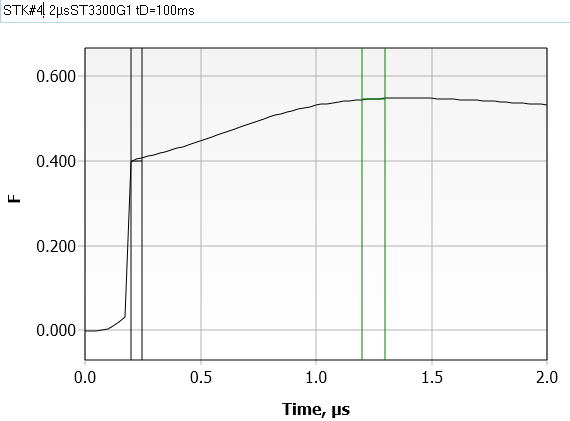
 **STK#4**


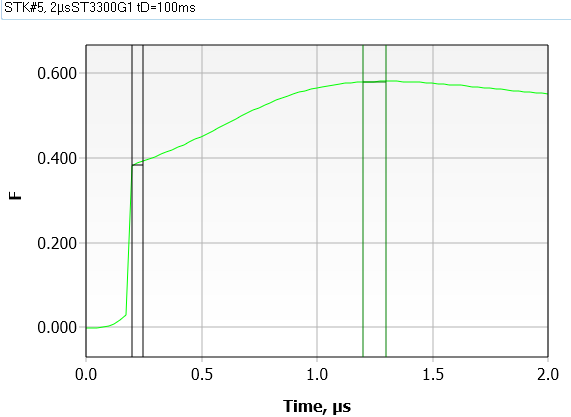
 **STK#5**


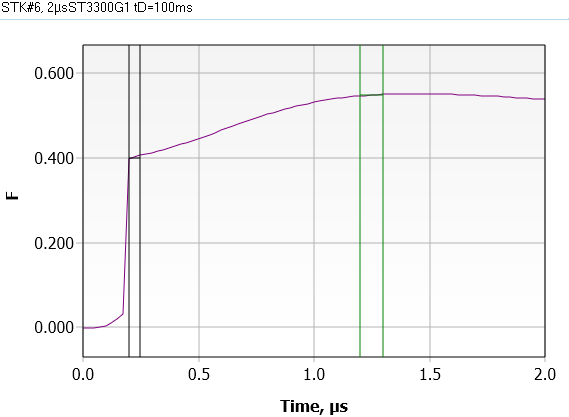
 **STK#6**

**S5 Assessment of the ST-induced changes of fluorescence yield using weak pulse-modulated measuring light**

As already outlined under Supplementary Materials S3, with the new device it is possible to carry out *combined* STK and PAM measurements by triggering an ST that is generated by the new ST-lamp at a defined point of time in a PAM Fast Kinetics recording (“embedded STK”). In this way, the time dependent effect of an ST generated by the ST-lamp can be assessed with the help of weak PAM ML. This is similar but *more informative* than the classical pump-and-probe approach, where a single weak probe flash is applied to determine the fluorescence yield at a defined dark-time following a pump flash. Using the repetitively applied 1µs PAM ML pulses, with a single run the *whole time course of the relaxation kinetics* is obtained. In the below screenshot the relaxation kinetics measured with a dilute suspension of dark-adapted *Chlorella* in the absence (red trace) and presence of 10µM DCMU (blue trace) are compared. A 3µs ST at 100% intensity of the ST-lamp (3300 mV supply voltage) was triggered at t=0. It may be noted that the *maximal* flash-induced fluorescence yield (F_m_^ST^) can be assessed *in the presence of DCMU only*, when ST-induced non-photochemical quenching (HIQ) is fully relaxed before photochemical quenching due to Q_A_ reoxidation (via the back reaction) develops. In the control, Q_A_ reoxidation overlaps with HIQ relaxation in the 0-200µs range following the ST, with the resulting peak being distinctly lower than the F_m_^ST^ measured in presence of DCMU.


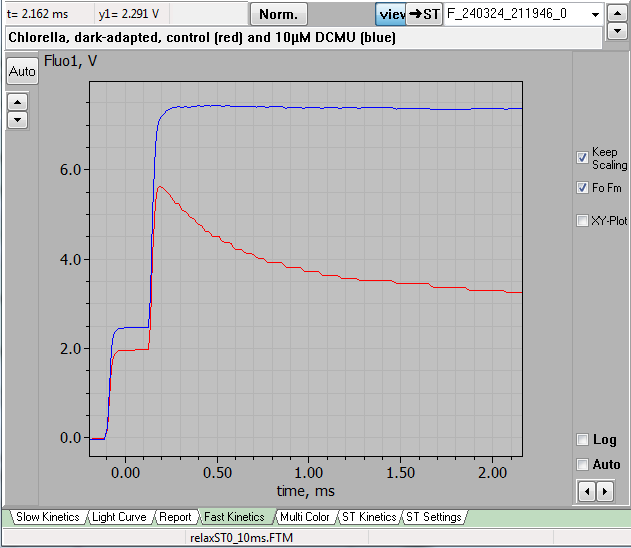


3µs ST at 100% intensity triggered at time=0 of PAM Fast Kinetics recording.

Dilute suspension of *Chlorella* in the absence (red) and presence of 10µM DCMU (blue).

Frequency of pulse-modulated ML programmed to decrease from 100 KHz to 10 KHz starting 100µs after the ST.

Differently from the ST provided by the standard Multi-Color Emitter unit, the quantum flux density generated by the new ST-lamp can be readily varied via the supply voltage, which can be defined under software control (see Supplementary Materials S2). For the experiment of Fig. 12 (main text) a Script was written for automated combined STK and PAM Fast Kinetics measurements at ten values of of supply voltages, resulting in 5, 10, 15, 20, 25, 30, 40, 60, 80 and 100% of maximal intensity. The same Script was used for the low fluence range (with maximal intensity attenuated to 5% with the help of Lee #209 plus Lee#211 neutral density filter foils placed between the cuvette and the perspex light guide connecting with the ST-lamp) and for the high fluence range without attenuation by neutral density filter foils.

It is an important advantage of this approach that the increases of fluorescence yield (∆F) that are induced by ST pulses of largely different intensities, is assessed at constant intensity of the pulse-modulated measuring light. Without the need to correct for the profile of ‘probe pulses’, the PAM signals allow to determine the ∆F directly from the Fast kinetics recordings. No normalization is required. In this way, the relationship between ST intensity and ∆F (saturation curve) can be measured very reliably and reproducibly. The below screenshot shows a selection of superimposed original recordings that were measured for the saturation curve displayed in Fig. 12 (main text).


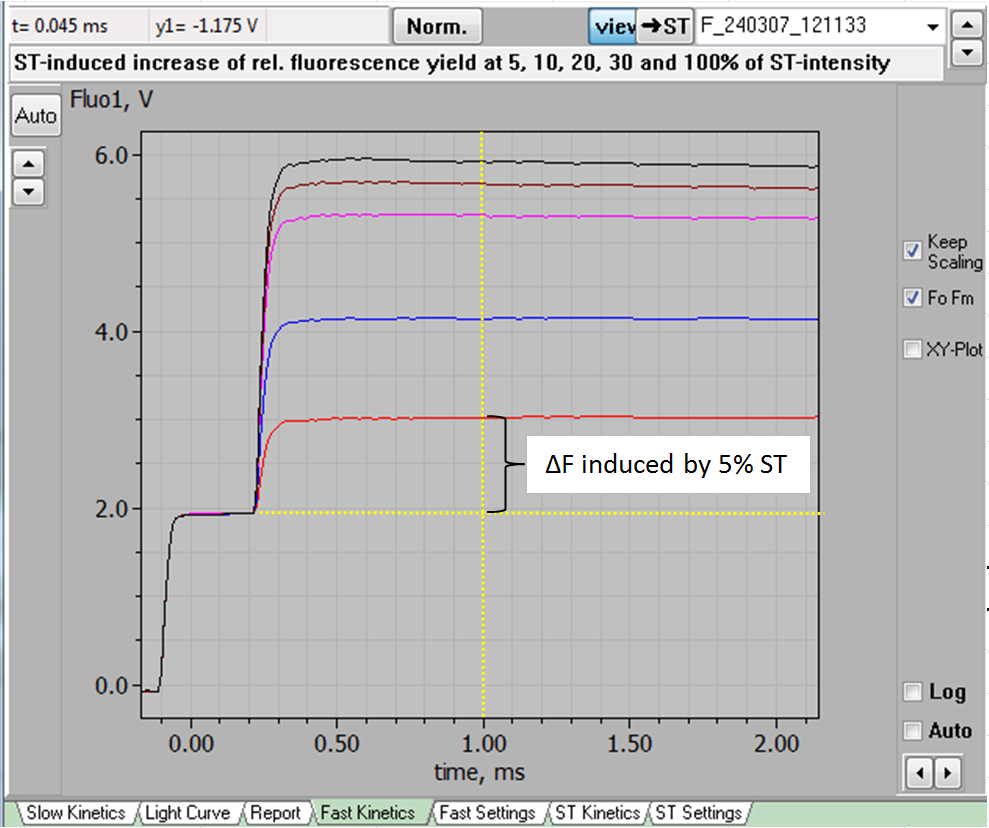


3µs ST triggered at time=0 of PAM Fast Kinetics recording at the following intensities:

5% (red), 10% (blue), 20% (magenta), 30% (brown) and 100% (black).

Dilute suspension of *Chlorella* in the presence of 10µM DCMU.

Frequency of pulse-modulated ML programmed to decrease from 100 KHz to 10 KHz starting 100µs after the ST.


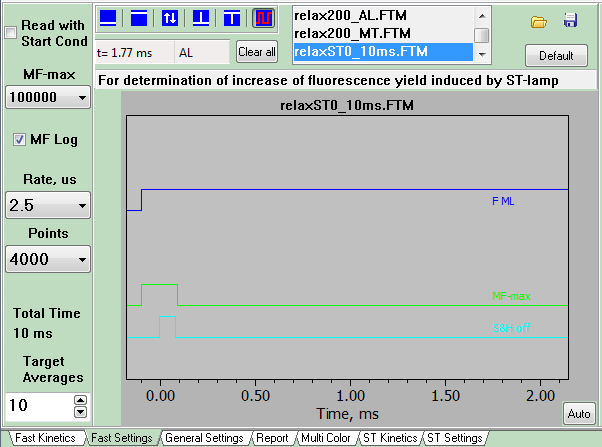


Trigger program for Fast Kinetics recording, with which the ST-induced ∆F was measured. The ST generated by the new ST-lamp was triggered at time=0. Pulse modulated ML was switched on at -100µs, together with maximal pulse frequency (100KHz).

**S6 Analysis of the “low-light effect” on F_0_ and period-4 oscillations**

In Figs. 8, 13 and 14 (main text) it was shown that the changes in fluorescence yield induced by ST pulses are considerably modified by weak FR background light. In particular, the change of the period-4 oscillation patterns of F_0_ and F_m_^ST^ in a series of ST-Kinetics (STKS) suggests that weak FR background illumination causes S-state advancement by one step. As FR (730-740nm) is absorbed more efficiently in PSI than in PSII, it appears reasonable to assume that the observed effect is driven via PSI activity. This assumption, however, may be questioned by the observation, that a very similar S-state advancement can also be induced with weak 540nm background illumination, as documented by the following screenshots.


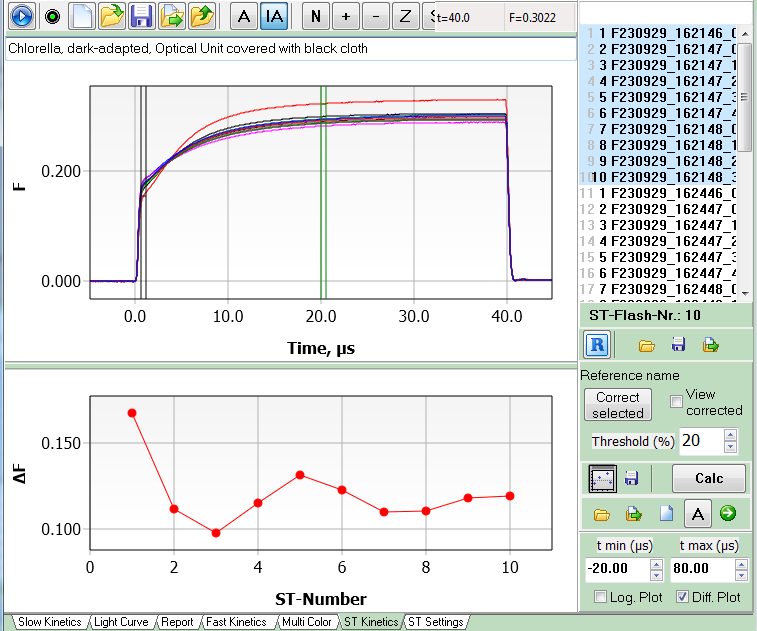
 **dark control**


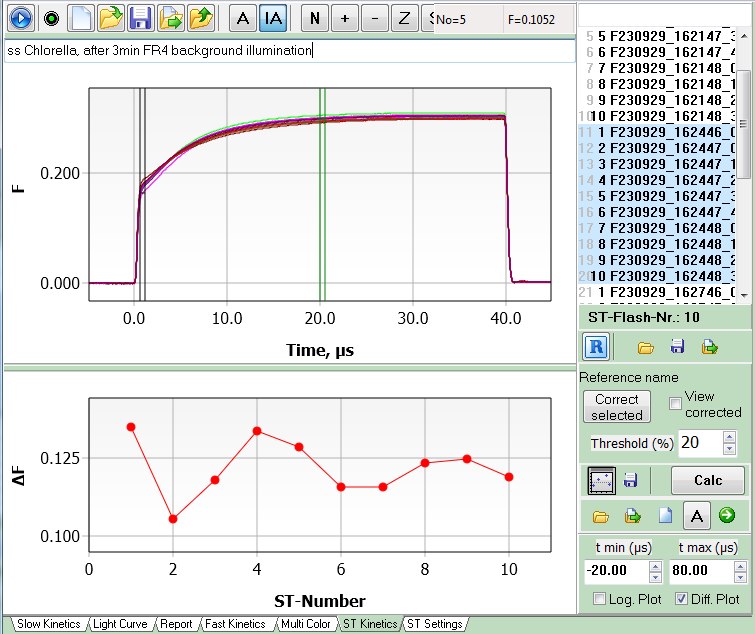
 **2µmol/(m²s) FR background**


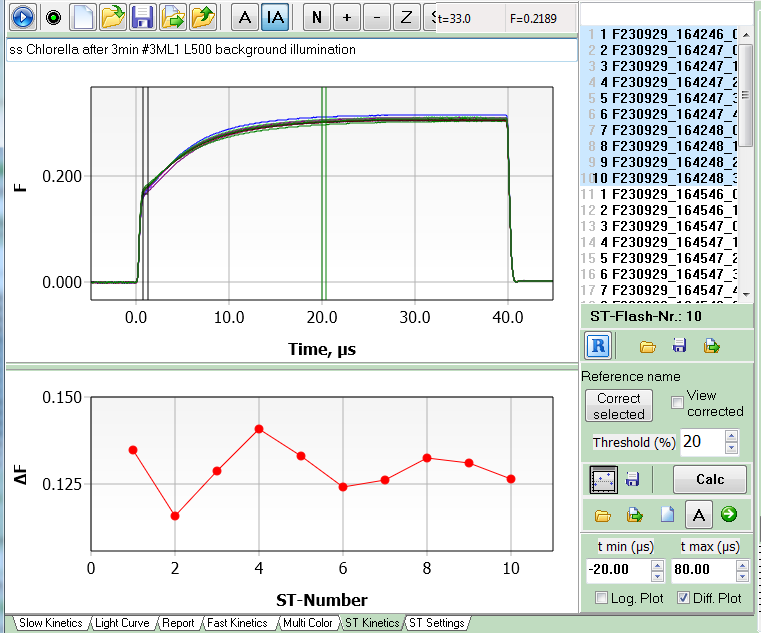
 **0.23 µmol/(m²s) 540nm background**

While the observed shift in the period-4 oscillation pattern clearly indicates that both types of weak background light strongly influence the initial S-state distribution in PSII, the molecular mechanism of this effect is uncertain. In particular, it is not clear whether it is driven by PSII or PSI action. The following hypothetical mechanisms in favor of PSII or PSI may be considered:

**Possible mechanism involving PSII action:** If after thorough dark adaptation the S-state distribution would contain a substantial population of S_0_, this could be *directly* transformed via PSII action into S_1_ by both weak FR and 540nm background illumination, when sufficient time is given. While weak background light will also lead to transient formation of S_2_ and S_3_, these states are likely to be deactivated again during the long dark time before the next quantum for charge separation arrives.

**Possible mechanism involving PSI action:** Both FR and 540nm light can influence the state of PSII components *indirectly* via PSI action by *oxidation of the PQ-pool*. The primary and secondary PSII acceptors, Q_A_ and Q_B_ are in equilibrium with the PQ-pool. Furthermore, S-state deactivation in the dark depends on electron donation from outside PSII, i.e. most likely from stroma reductants that reduce the PQ-pool. Hence, it appears feasible that the dark S-state distribution *in vivo* is controlled by the redox state of the PQ pool, which again is determined by the stroma reducing power and can be shifted towards oxidation by controlled PSI action.

Considering the complexity of *in vivo* photosynthesis, to obtain unequivocal experimental evidence in favor of PSII or PSI action being responsible for the observed “low light effect” is a difficult task. For obtaining clear-cut evidence in favor of one or the other of the proposed mechanisms, a dedicated separate investigation is required. Here we just intend to demonstrate the tools that are provided by the new device to tackle this task. For this purpose, on one hand it is a great advantage that PAM measurements allow to assess the quasi-dark state (i.e. non-intrusively, without any significant influence on the fluorescence yield) over extended periods of time (minutes and even hours) via Slow Kinetics recordings. On the other hand, via flash-kinetics (STK) and flash-train (STKS) measurements, the state of PSII (including the S-state distribution) can be analysed. Below some preliminary data are presented that demonstrate the complementarity of the two approaches.

**STK embedded in Slow Kinetics recordings of the fluorescence changes induced in the vicinity of F_0_ by weak FR and weak 540nm light**

As shown in Figs. 13 and 14 (main text) and also apparent from the screenshots presented above, weak FR background illumination results in a small increase of “dark” fluorescence yield that accompanies the FR induced S-state advancement apparent in the STKS recording. At minimal intensity and frequency of 540nm ML it is possible to measure the quasi-dark fluorescence yield over extended periods of time (even hours) via PAM Slow Kinetics recordings and to evaluate the changes that are induced by FR or 540nm light. The below screenshot shows a recording (running over almost 50min) with a continuously stirred dilute suspension of *Chlorella* that before the experiment was thoroughly dark-adapted.


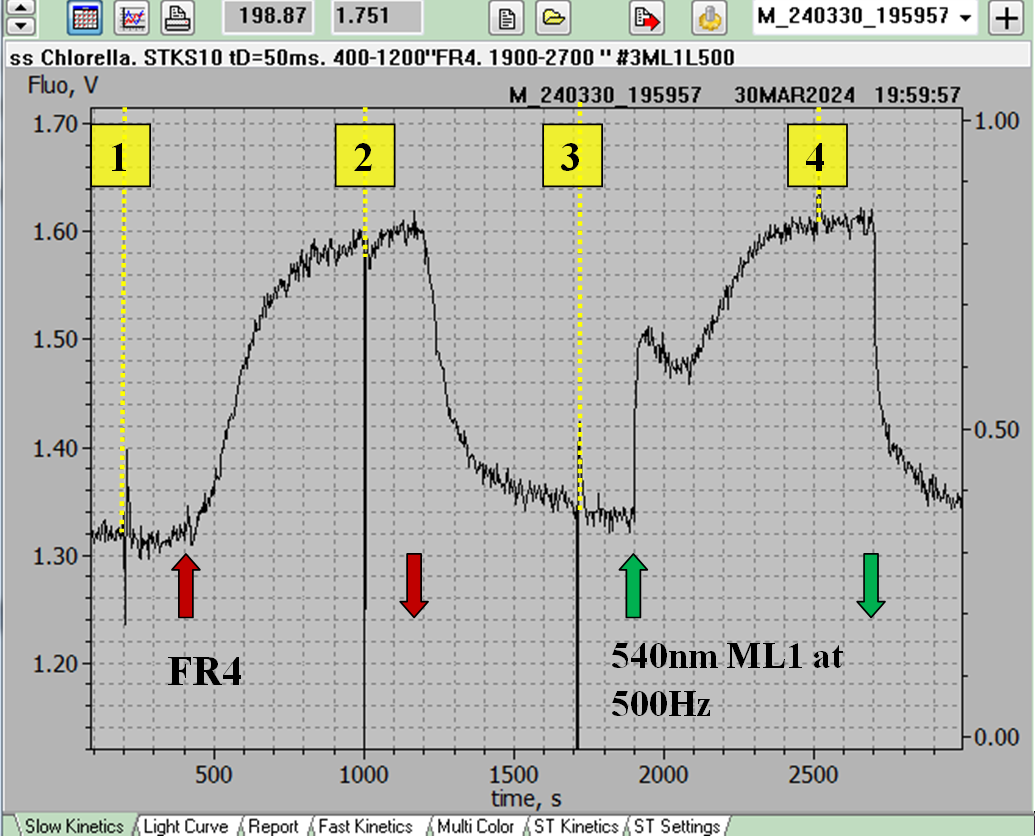


Comparison of the changes of “dark” fluorescence yield induced by weak FR light (2 µmol 730-740nm quanta m^-2^s^-1^) and weak green light (0.23 µmol 540nm quanta m^-2^s^-1^).

Where indicated by the yellow lines, STKS recordings were “embedded” into the PAM Slow Kinetics recordings for assessment of the state of PSII. The period-4 oscillation patterns observed in the four different states of PSII (marked by yellow lines) are presented in the following screenshots.

The FR induced fluorescence response revealed by the PAM Slow Kinetics displays a very minor rapid step (about 5% of total increase) and then shows a sigmoidal slow rise phase with a half-time of about 3 min. In contrast, the fluorescence rise induced by the weak 540nm light displays a very pronounced rapid step (about 60% of total increase), followed by a transient decline (lasting about 2 min) and a second, slow sigmoidal rise phase, which resembles the main FR induced increase.

Based on analogous measurements using higher FR intensities (not shown in the figures) it may be assumed with some certainty that the depicted *FR induced* increase of fluorescence yield is mainly due to a state 2 to state 1 transition. This transition sets in when the PQ-pool (partially reduced in the dark) becomes reoxidized via PSI activity. In the case of illumination with weak 540nm light, fluorescence first rapidly responds to partial Q_A_ reduction driven by PSII, most likely reflecting “inactive PSII” (vacant Q_B_ site). The following dip-phase presumably reflects PSI driven oxidation of the PQ pool, which “triggers” a similar state 2 to state 1 transition as with FR illumination. We note that the dominant rapid fluorescence increase induced by weak 540nm is mostly missing in the FR response, suggesting that the applied 540nm light excites PSII much more efficiently than the applied FR. In view of the similar S-state advancement induced by FR and 540nm, this may argue against the hypothesis that the FR effect is driven by PSII action.


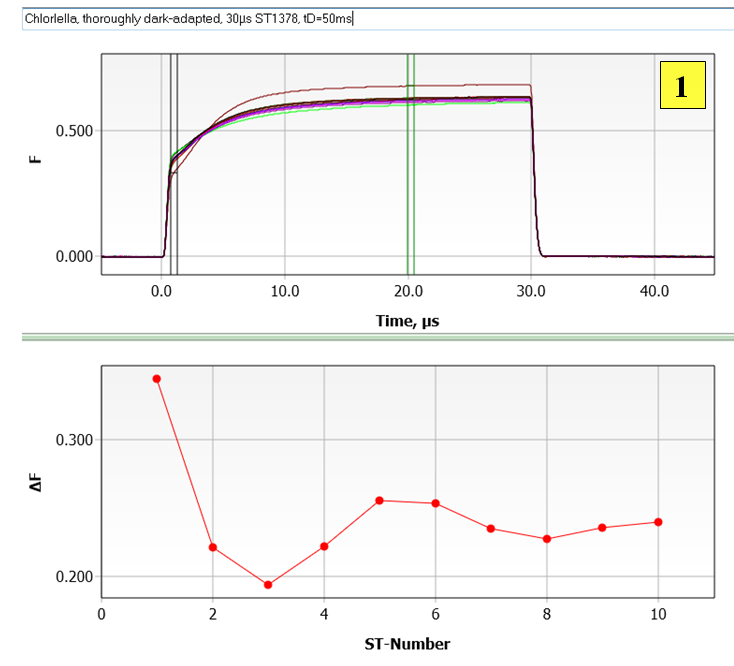


**Measured after thorough dark-adaptation (Optical Unit covered with black cloth to avoid “traces” of ambient light).**

**Minimum with ST#3,**

**Maximum with ST#5**


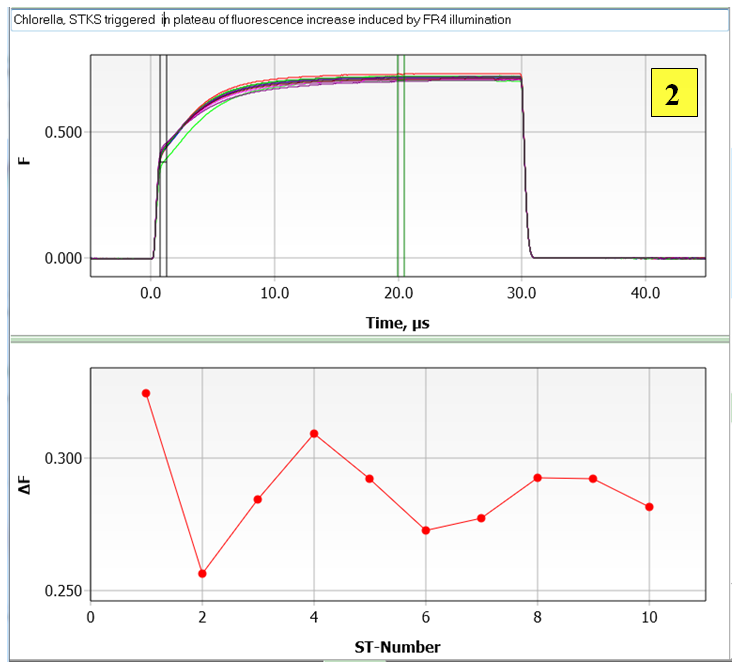


**Measured in plateau of fluorescence increase reached after 10 min illumination with 2µmol/(m²s) 730-740nm light.**

**Minimum with ST#2,**

**Maximum with ST#4**


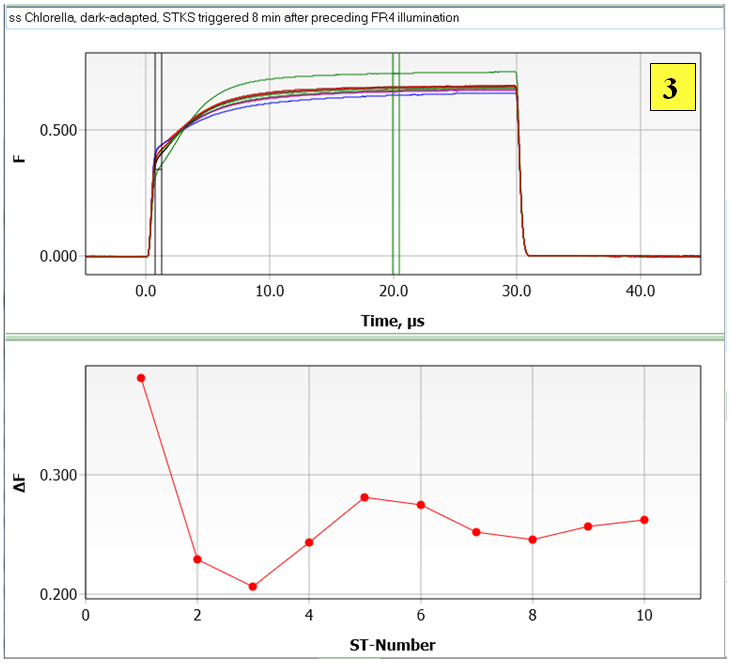


**Measured after the preceding FR-illumination and STKS-recording with 8 min dark-adaptation.**

**Minimum with ST#3,**

**Maximum with ST#5**


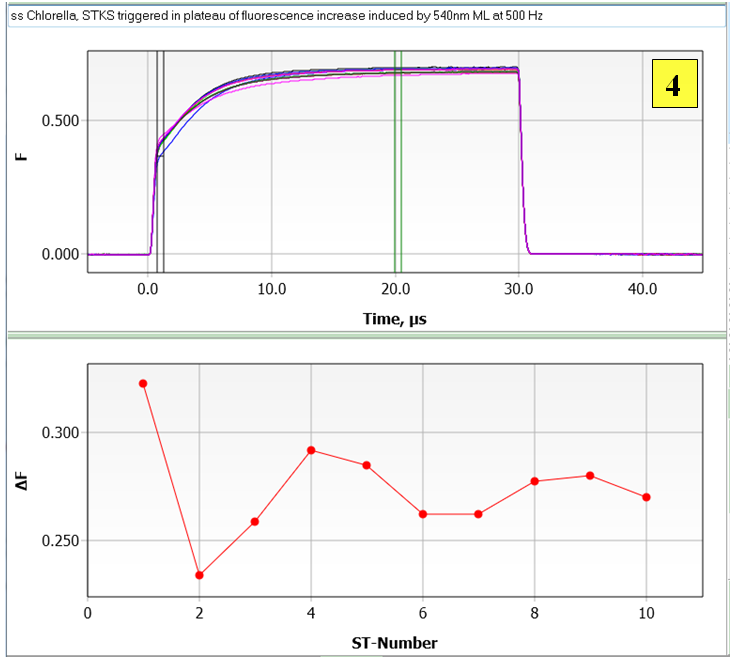


**Measured in plateau of fluorescence increase reached after 10 min illumination with green light (0.23 µmol 540nm quanta m^-2^s^-1^).**

**Minimum with ST#2,**

**Maximum with ST#4**

In the case of the 540nm induced S-state advancement, the associated 540nm induced fluorescence increase is quite complex and the question may be asked, which of the reactions underlying the various phases of this increase is mostly responsible for the S-state advancement. An answer to this question is given by the measurements presented in the following screenshots.


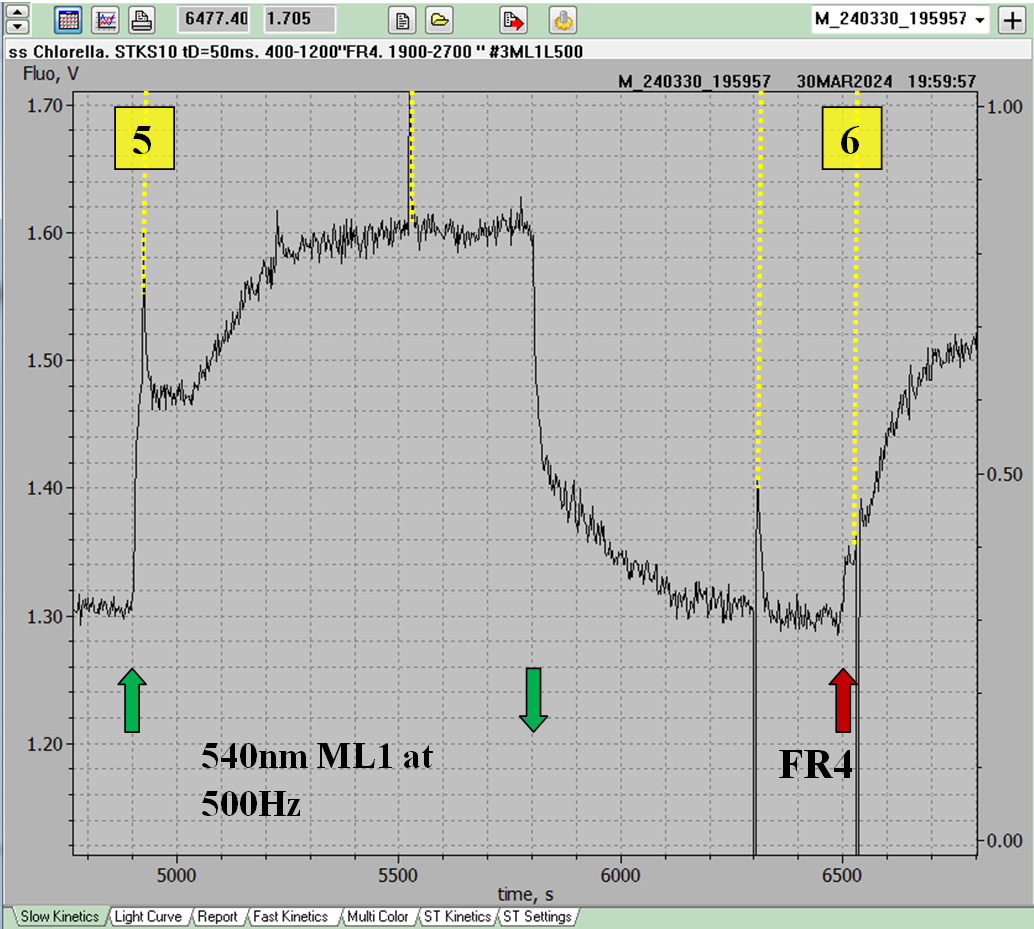


Comparison of the changes in fluorescence yield induced by weak green light (0.23 µmol 540nm quanta m^-2^s^-1^) and weak FR light (2 µmol 730-740nm quanta m^-2^s^-1^)

Where indicated by the yellow lines, STKS recordings were “embedded” into the PAM Slow Kinetics recordings. The resulting period-4 oscillations of fluorescence yield are displayed below.


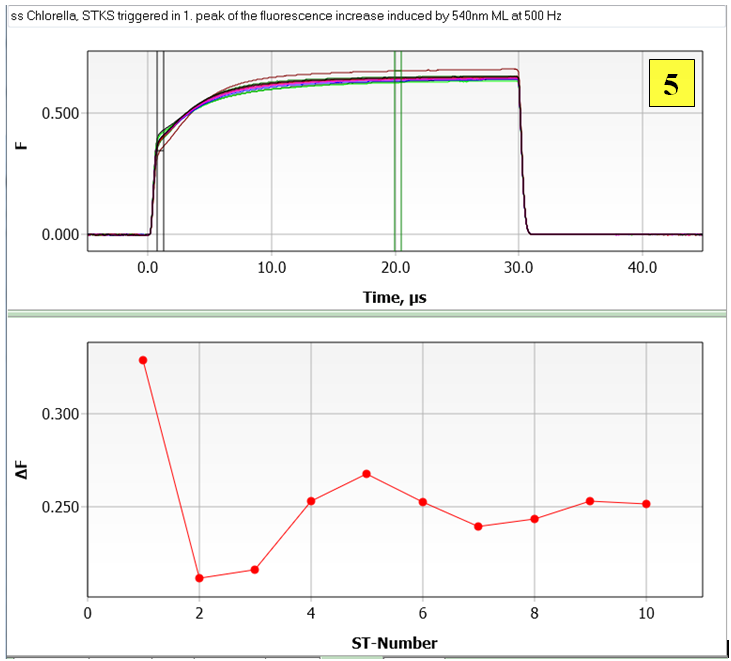


**Measured at the end of the rapid first step of fluorescence increase induced by weak green light (0.23 µmol 540nm quanta m^-2^s^-1^)**

**Minimum with ST#2,**

**Maximum with ST#5**

When the STKS is triggered in the first peak of the 540nm induced fluorescence increase, i.e. at the end of the rapid PSII driven phase, the STK maximum is still with STK#4, i.e. as if the sample were still dark-adapted).


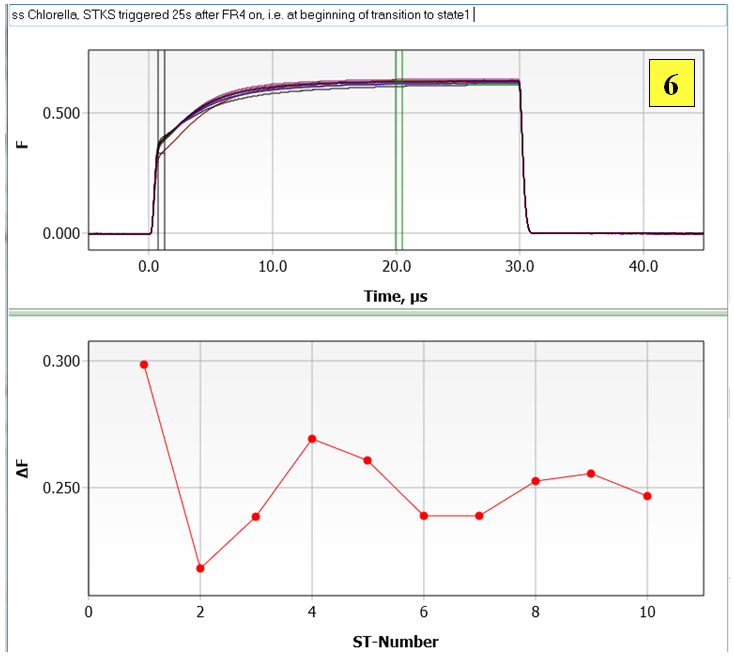


**Measured 25s after onset of weak FR illumination (2 µmol 730-740 nm quanta m^-2^s^-1^) at the beginning of the sigmoidal increase of fluorescence yield.**

**Minimum with ST#2,**

**Maximum with ST#4**

When the STKS is triggered 25s after FR4 on, i.e. at the beginning of the slow fluorescence rise caused by the transition from state 2 to state 1, the period-4 oscillation pattern already shows a clear-cut S-state advancement compared to the dark-adapted state. This means that the decisive reaction for the change in S-state distribution already has taken place before the state transition. A common prerequisite for state 1 formation and S-state advancement is an oxidized PQ-pool.

In conclusion, the presented data support the hypothesis that the S-state advancement is driven by PSI activity via oxidation of the PQ-pool, which *in vivo* is partially reduced in dark-adapted samples.

In analogous measurements with *Chlorell*a as well as with *Synechococcus leopoliensis*, the reducing power of stroma reductants was varied via extended periods of continuous illumination at different intensities. These experiments revealed an intriguing interplay between PSI driven cyclic electron flow, chlororespiratory electron flow, reversible state 1-state 2 transitions and the states of PSII donor and acceptor sides *in vivo*, the complexity of which forbids further discussion in the present Emerging Techniques communication. We want to point out, however, that this kind of *in vivo* measurements may be foreseen to constitute an important field of future applications of the new instrument.
